# Association of Prenatal Care Services, Maternal Morbidity, and Perinatal Mortality With the Advanced Maternal Age Cutoff of 35 Years

**DOI:** 10.1001/jamahealthforum.2021.4044

**Published:** 2021-12-03

**Authors:** Caroline K. Geiger, Mark A. Clapp, Jessica L. Cohen

**Affiliations:** 1 Harvard University, Interfaculty Initiative in Health Policy, Cambridge, Massachusetts; 2 Evidence for Access, Genentech Inc, South San Francisco, California; 3 Department of Obstetrics and Gynecology, Massachusetts General Hospital, Boston; 4 Department of Global Health and Population, Harvard T.H. Chan School of Public Health, Boston, Massachusetts

## Abstract

**Importance:**

Maternal and perinatal mortality remain high in the US despite growing rates of prenatal services and spending, and little rigorous evidence exists regarding the impact of prenatal care intensity on pregnancy outcomes. Patients with an expected date of delivery just after their 35th birthday may receive more intensive care owing to the advanced maternal age (AMA) designation; whether this increase in prenatal care is associated with improvements in outcomes has not been explored.

**Objective:**

To determine the association between the AMA designation and prenatal care services, severe maternal morbidity, and perinatal mortality.

**Design, Setting, and Participants:**

This cross-sectional study used a regression discontinuity design to compare individuals just above vs just below the 35-year AMA cutoff, using unidentifiable administrative claims data from a large, nationwide commercial insurer. All individuals with a delivery between January 1, 2008, and December 31, 2019, who were aged 35 years within 120 days of their expected date of delivery were included in the study. Analyses were performed from July 1, 2020, to February 1, 2021.

**Exposures:**

Individuals who were aged 35.0 through 35.3 years on the expected date of delivery were designated as AMA.

**Main Outcomes and Measures:**

Outcomes were visits with specialists (obstetrician-gynecologists and maternal-fetal medicine), ultrasound scan use, antepartum fetal surveillance, aneuploidy screening, severe maternal morbidity, preterm birth or low birth weight, and perinatal mortality.

**Results:**

The analysis included 51 290 individuals (mean [SD] age; 34.5 [0.5] years); 26 108 individuals (50.9%) were aged 34.7 to 34.9 years and 25 182 individuals (49.1%) were aged 35.0 to 35.3 years on the expected date of delivery. A total of 2407 pregnant individuals (4.7%) had multiple gestation, 2438 (4.8%) had pregestational diabetes, 2265 (4.4%) had chronic hypertension, and 4963 (9.7%) had obesity. Advanced maternal age was associated with a 4.27 percentage point increase in maternal-fetal medicine visits (95% CI, 2.27-6.26 percentage points; *P* < .001), a 0.21 unit increase in total ultrasound scans (95% CI, 0.06-0.37; *P* = .006), a 15.67 percentage point increase in detailed ultrasound scans (95% CI, 13.68-17.66 percentage points; *P* < .001), and a 4.86 percentage point increase in antepartum surveillance (95% CI, 2.83-6.89 percentage points; *P* < .001). The AMA designation was associated with a 0.39 percentage point decline in perinatal mortality (95% CI, −0.77 to −0.01 percentage points; *P* = .04).

**Conclusions and Relevance:**

In this cross-sectional study, the AMA designation at age 35 years was associated with an increase in receipt of prenatal monitoring and a small decrease in perinatal mortality, suggesting that the AMA designation may be associated with clinical decision-making, with individuals just older than 35 years receiving more prenatal monitoring. These results suggest that increases in prenatal care services stemming from the AMA designation may have important benefits for fetal and infant survival for patients in this age range.

## Key Points

**Question:**

What is the association between the advanced maternal age (AMA) cutoff of 35 years and prenatal care service intensity, severe maternal morbidity, and perinatal mortality?

**Findings:**

In this cross-sectional study of 51 290 deliveries, using regression discontinuity methods, the AMA designation was associated with a significant increase in prenatal care services, including prenatal visits, ultrasound scans, and antepartum surveillance. The AMA designation was associated with a large decline in perinatal mortality but not severe maternal morbidity.

**Meaning:**

Results suggest that increases in prenatal care intensity associated with the commonly applied AMA designation may have important benefits for perinatal survival for patients aged approximately 35 years.

## Teaser

This cross-sectional study investigates the association between the advanced maternal age cutoff of 35 years and prenatal care service intensity, severe maternal morbidity, and perinatal mortality.

## Introduction

Reducing maternal and newborn morbidity and mortality in the US is a critical priority.^1,2^ Maternal mortality and severe morbidity are higher in the US than in other high-income countries, with 20.1 maternal deaths per 100 000 live births in 2019.^2,3,4^ In addition, stillbirth rates in the US remain high at 3 per 1000 births and have been stagnant for years.^5^ The Healthy People 2030 goals identify reductions in perinatal morbidity as a key priority.^6^ Despite the policy commitment to improving the accessibility and quality of prenatal care, rigorous evidence regarding the effect of prenatal care services on maternal and newborn outcomes is inadequate.^6,7,8^

High-quality prenatal care is a widely recognized determinant of pregnancy outcomes. Clinical guidelines recommend at least 12 prenatal visits and 2 ultrasound scans, but many patients receive considerably more intensive monitoring and testing.^9,10^ For example, fetal surveillance, genetic testing, and use of maternal-fetal medicine (MFM) specialists are commonly provided services aimed at preventing stillbirth and adverse newborn outcomes.^11,12,13^ Although prenatal visits, testing, and screening have all been increasing, considerable uncertainty exists about how the intensity of prenatal services translates into pregnancy outcomes.^7,14^ Much of the content of prenatal care guidelines has persisted for decades without strong causal evidence to demonstrate its value.^7,8^

Strong evidence on the link between prenatal care intensity and pregnancy outcomes is difficult to generate because patients who receive more intensive prenatal care are different from those who do not in many ways that can confound this relationship.^15^ For example, patients with more prenatal monitoring are more likely to have underlying risk factors and to be of higher socioeconomic status, both of which influence the risk of poor pregnancy outcomes.^15,16^ Randomized clinical trials with pregnant patients are rare and typically focus on new interventions.^7^

In this study, we leverage the obstetrical designation of advanced maternal age (AMA), defined as an age of 35 years or older on the expected date of delivery, to explore the association between prenatal care intensity and maternal and newborn outcomes using regression discontinuity (RD) methods. Maternal age is a recognized risk factor for multiple adverse pregnancy outcomes, including maternal morbidity and stillbirth, and, as such, may influence clinician decision-making and recommendations for the use of prenatal care services aimed at mitigating risk (eg, visit frequency and antepartum fetal surveillance).^11,17^ However, although pregnancy-related risks increase with maternal age, there is no known abrupt biological increase in underlying risk precisely at age 35 years.^18^ The 35-year threshold was set in 1979 as a guideline for routinely offering invasive testing for trisomy 21, but has since been extrapolated to other maternal and fetal risks and is now commonly used to justify increased antenatal screening.^19,20,21^ Furthermore, payers may reinforce how this designation is used in clinical obstetrics, eg, through the use of AMA in billing guidelines as an indication for prenatal services such as cell-free DNA testing.^22^

Using administrative claims data from a sample of more than 50 000 commercially insured pregnant patients, our RD approach looked for abrupt discontinuities in prenatal care services and pregnancy outcomes at the 35-year cutoff. Because pregnant patients with due dates within a few months before vs after their 35th birthday should not differ meaningfully—other than their likelihood of receiving more prenatal care services owing to the AMA designation—patients within this narrow age bandwidth can be thought of as effectively randomized to the AMA designation. We explore whether prenatal care services increase at the AMA designation threshold and whether this increase in prenatal services is associated with changes in severe maternal morbidity and perinatal mortality.

## Methods

### Data

The primary data source for this cross-sectional study was deidentified administrative claims data from a large commercial insurer. The data included medical claims and monthly enrollment details for more than 60 million individuals from January 1, 2008, to December 31, 2019, and each individual’s exact date of birth. Billed medical claims and encounter data were used to identify services provided before and during childbirth. This study of deidentified data was deemed exempt by the Harvard University Longwood Campus institutional review board. This study followed the Strengthening the Reporting of Observational Studies in Epidemiology (STROBE) reporting guideline.

### Sample

The sample included all pregnant people with a delivery covered by the insurer who were within 120 days of turning age 35 years on the expected date of delivery (eTable 1 in the Supplement). As information regarding race and ethnicity was not available in the claims data, it was not analyzed in this study. However, we included variables that were percentage Hispanic individuals and percentage White individuals in the zip code based on publicly available summary data from the American Community Survey. No other race or ethnicity was included as a zip-level variable because of the high rates of missing data in the American Community Survey due to suppression in small zip codes with low diversity.

The age on the expected date of delivery was calculated using the patient’s exact birth date and the estimated expected date of delivery based on the infant’s gestational age on the actual date of delivery (eTable 2 in the Supplement). Inclusion criteria included continuous enrollment during the entire pregnancy, a zip code of residence, and at least 1 outpatient encounter and 1 ultrasound scan during pregnancy.

### Outcomes

Our primary outcomes were severe maternal morbidity, as defined by the Centers for Disease Control and Prevention and measured during pregnancy through 6 weeks postpartum; and perinatal mortality, which is defined by the National Center for Health Statistics as a fetal death at 28 weeks’ gestation or later or neonatal death within 7 days after delivery.^23,24,25^ We were unable to measure maternal deaths in our data. We included whether the infant was born preterm (<37 weeks’ gestation) or with low birth weight (<2500 g) as a measure of newborn morbidity.

Our measures of prenatal care services included the number of obstetrician-gynecologist (OBGYN) visits, any MFM specialist visit, the total number of ultrasound scans, any detailed ultrasound scan, any antepartum fetal surveillance (ie, biophysical profile and/or nonstress test), and any aneuploidy screening (ie, serum analyte screening, cell-free DNA, and/or invasive genetic testing) (eTable 3 in the Supplement). Consistent with other national insurers, this insurer included AMA as one of multiple indications in the billing guidelines for cell-free DNA testing, antepartum surveillance, and detailed ultrasound scans.

### Statistical Analysis

We used an RD design to analyze the association between the 35-year age cutoff and the outcomes of interest. Regression discontinuity overcomes the issue of selection bias in comparing individuals with and without an intervention by exploiting an arbitrary cutoff in clinical decision-making rules or program eligibility, providing transparent visual evidence of changes in the outcome at the cutoff.^26,27,28,29^ The RD method relies on the assumption that individuals within a narrow bandwidth around the cutoff have similar baseline characteristics other than the likelihood of receiving the intervention. Although the risk of adverse outcomes increases with maternal age, individuals 4 months older or younger than 35 years should not have different underlying risks. There should be no reason to expect abrupt discontinuities in outcomes at age 35 years, other than those stemming from differences in care due to the AMA designation.

We used local linear regression, with a binary indicator for age 35 years or older on the expected date of delivery as the main independent variable. Regressions controlled for the total number of days between the individual’s 35th birthday and the expected date of delivery (the running variable), prepregnancy characteristics (ie, pregestational diabetes, chronic hypertension, obesity, and multiple gestation), zip code characteristics (percent Hispanic individuals, percent White individuals, median household income, and urbanicity), and county of residence characteristics (any hospital with a neonatal intensive care unit and OBGYNs per 10 000 deliveries), with fixed effects for state of residence, year, and month of delivery to improve precision. All regressions were estimated using a bandwidth of 120 days around the cutoff with triangular kernel weights, which give more weight to observations closer to the cutoff and are recommended for RD analyses.^30^ Because we could only measure gestational age in weeks, we used a “donut hole” RD, excluding individuals with an expected date of delivery within 7 days of their 35th birthday.^31^ We plotted outcomes by the running variable to visually inspect the discontinuities in the outcome at the 35-year cutoff. Although many RD designs can be used as a form of instrumental variable analysis, in which the threshold is used as an instrument in a “fuzzy RD” framework, we did not take this additional step in this analysis.^26,28^ This is because the AMA cutoff can affect perinatal and maternal outcomes through multiple channels, some of which we were able to measure with our data and some we could not. Thus, an instrumental variable analysis would not necessarily meet the exclusion restriction for instrumental variable analysis, and estimates could be biased.

#### Subgroup Analysis

We conducted a subgroup analysis among individuals with a low-risk pregnancy, defined as singleton gestation and no pregestational diabetes, chronic hypertension, or obesity (eTable 3 in the Supplement). Because these individuals were less likely to have indications for additional prenatal care services separate from age, changes in prenatal care services at the AMA cutoff were expected to be even larger than those of the full sample.^11,32^

#### Sensitivity Analyses

Following the RD literature, we conducted a number of sensitivity analyses and falsification tests. First, we tested whether the total number of deliveries was smooth across the 35-year cutoff.^33^ Second, we tested for changes in maternal, infant, and zip code or county characteristics across the cutoff. Third, we tested for changes in the likelihood of a pregnancy ending in termination or miscarriage at the cutoff. Fourth, we showed that the results were not sensitive to bandwidth selection or covariate inclusion. Fifth, we showed that results were unique to the 35-year cutoff by running placebo tests using cutoffs above and below 35 years. Finally, we verified that clinicians were in fact designating AMA based on age at the calculated expected date of delivery by testing for increases in “elderly primigravida and/or multigravida” diagnosis codes at the cutoff.

Full details of all sensitivity analyses are provided in eTables 6 to 13 and eFigure 5 in the Supplement. Analyses were implemented using R software, version 3.6.1 (R Foundation). Local linear regressions were implemented using the “rdd” package. All *P* values were 2-sided, and a threshold of *P* < .05 was used for statistical significance. Statistical analyses were performed from July 1, 2020, to February 1, 2021.

## Results

### Sample Characteristics

A total of 51 290 individuals (mean [SD] age; 34.5 [0.5] years) met all sample selection criteria and had an expected date of delivery within 120 days of their 35th birthday (eTable 4 in the Supplement). A total of 26 108 (50.9%) individuals were aged 34.7 to 34.9 years on the expected date of delivery and just below the AMA cutoff, and 25 182 individuals (49.1%) were aged 35.0 to 35.3 years on the expected date of delivery. A total of 2407 pregnant individuals (4.7%) had multiple gestation, 2438 (4.8%) had pregestational diabetes, 2265 (4.4%) had chronic hypertension, and 4963 (9.7%) had obesity (Table 1).

**Table 1.** Characteristics of Individuals Within 120 Days of Age 35 Years on the Expected Date of Delivery^a^

Characteristics	Age at expected date of delivery, No. (%),^b^ y	
	34.7-34.9	35.0-35.3
Total deliveries, No.	26 108	25 182
Maternal characteristics		
Pregestational diabetes	1226 (4.70)	1212 (4.81)
Chronic hypertension	1138 (4.36)	1127 (4.48)
Obesity	2529 (9.69)	2434 (9.67)
Multiple gestation	1198 (4.59)	1209 (4.80)
Prenatal care services		
Total OBGYN visits, mean (SD)	8.44 (6.25)	8.92 (6.43)
Any MFM visit	12 991 (49.76)	14 104 (56.01)
Any aneuploidy screening	19 448 (74.49)	19 328 (76.75)
Serum analyte	17 569 (67.29)	15 746 (62.53)
Cell-free DNA	3877 (14.85)	6871 (27.29)
Invasive test^c^	712 (2.73)	1065 (4.23)
Total ultrasound scan visits, mean (SD)	5.45 (3.88)	5.83 (4.07)
Any detailed ultrasound scan	11 270 (43.17)	16 195 (64.31)
Any antepartum fetal surveillance	13 539 (51.86)	14 507 (57.61)
Fetal nonstress test	10 664 (40.85)	11 454 (45.48)
Biophysical profile	7844 (30.04)	8643 (34.32)
Maternal and newborn outcomes		
Any severe maternal morbidity	922 (3.53)	851 (3.38)
Perinatal mortality	245 (0.94)	227 (0.90)
Preterm birth or low birth weight	3357 (12.86)	3376 (13.41)
Preterm birth	3143 (12.04)	3149 (12.50)
Low birth weight	2409 (5.96)	2418 (6.35)

Abbreviations: MFM, maternal-fetal medicine; OBGYN, obstetrician-gynecologist.

^a^ Sample includes all individuals with a delivery during the study period (2008-2019) who turned age 35 years within 120 days of the expected date of delivery. The expected date of delivery (assuming 40 weeks’ gestation) was defined based on the actual date of delivery and gestational age at delivery. Inclusion criteria were continuous eligibility during entire pregnancy period, a zip code of residence in the data, and also having at least 1 outpatient visit and 1 ultrasound scan during pregnancy. Individuals who turned age 35 years within 7 days of the expected date of delivery were excluded because gestational age was only measured in weeks in the data.

^b^ Data are presented as No. (%) unless otherwise specified.

^c^ Invasive genetic testing included amniocentesis or chorionic villus sampling.

### Prenatal Care Services

**Figure 1.**
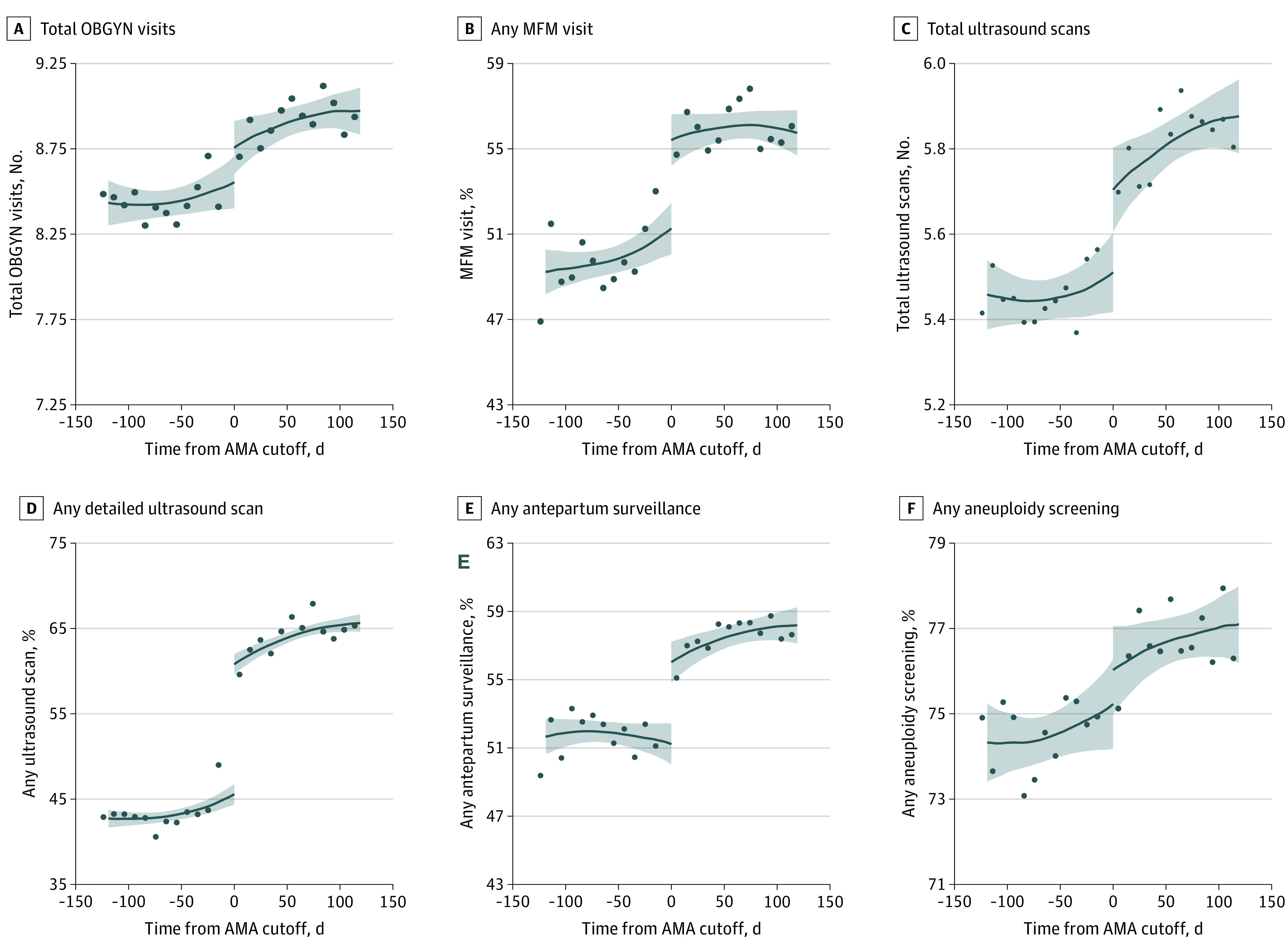
Prenatal Care Services by Weeks Relative to Age 35 Years on Expected Date of Delivery The figure shows adjusted local linear regression results for the regression discontinuity analyses (solid lines) and the 95% CIs (shaded areas) for total obstetrician-gynecologist (OBGYN) visits (A), any maternal-fetal medicine (MFM) visit (B), total ultrasound scans (C), any detailed ultrasound scan (D), any antepartum surveillance (E), and any aneuploidy screening (F). The points in the figure show the binned unadjusted outcomes plotted by the running variable (ie, the number of days between the expected date of delivery and 35th birthday). All panels show results for the full sample of deliveries with an expected date of delivery within 120 days of the 35th birthday (N = 51 290). As seen in the figure and the regression results, the advanced maternal age (AMA) designation was associated with significant increases in any MFM visits, total ultrasound scans, any detailed ultrasound scan, and any antepartum fetal surveillance but no significant changes in total OBGYN visits or aneuploidy screening.

Trends in prenatal care services around the 35-year age cutoff are presented in Figure 1. Although there was a visual increase in OBGYN visits at the AMA cutoff, the increase was not statistically significant. However, the proportion of individuals with any MFM visit increased by 4.27 percentage points (95% CI, 2.27-6.26 percentage points; *P* < .001; 8.1% change) at the AMA cutoff. Total ultrasound scans increased modestly (a 0.21 unit increase; 95% CI, 0.06-0.37; *P* = .006; 3.9% change), and there was a large 15.67 percentage point increase (95% CI, 13.68-17.66 percentage points; *P* < .001; 34.4% change) in any detailed ultrasound scan at age 35 years. Increases in any antepartum surveillance were also substantial, with an estimated 4.89 percentage point increase (95% CI, 2.83-6.89 percentage points; *P* < .001; 9.5% change) at age 35 years. There was no significant change in any aneuploidy screening at age 35 years; however, there was a significant shift from serum analyte screening toward cell-free DNA and invasive genetic tests at age 35 years (any serum analyte test declined, –4.02 percentage points; 95% CI, –5.94 to –2.09 percentage points; *P* < .001; any cell-free DNA test increased, 9.40 percentage points; 95% CI, 7.97-10.83 percentage points; *P* < .001; any invasive genetic test increased, 1.10 percentage points; 95% CI, 0.35-1.85 percentage points; *P* = .004) (eFigure 1 in the Supplement).

### Perinatal and Maternal Outcomes

**Figure 2.**
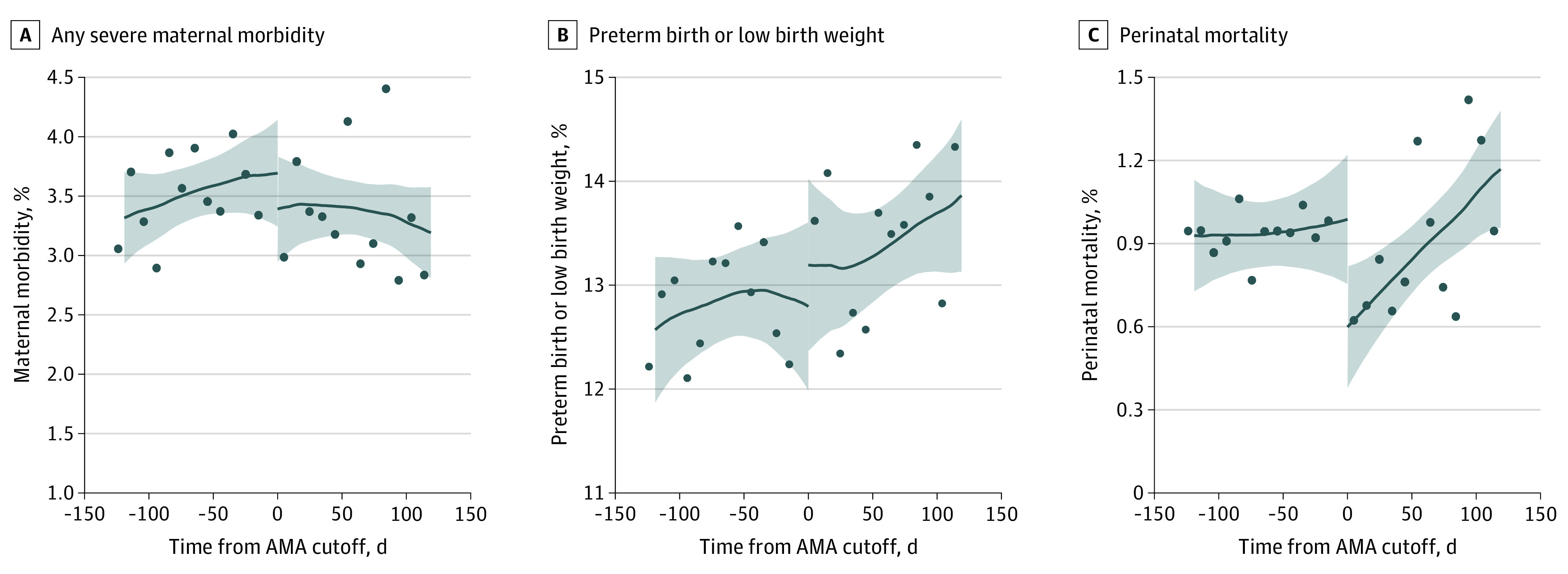
Maternal and Newborn Outcomes by Weeks Relative to Age 35 Years on Expected Date of Delivery The figure shows adjusted local linear regression results for the regression discontinuity analyses (solid lines) and the 95% CIs (shaded areas) for any severe maternal morbidity (A), preterm birth or low birth weight (B), and perinatal mortality (C). The points in the figure show the binned unadjusted outcomes plotted by the running variable (ie, the number of days between the expected date of delivery and 35th birthday). All figures show results for the full sample of deliveries with an expected date of delivery within 120 days of the 35th birthday (N = 51 290). As seen in the figure and the regression results, the advanced maternal age (AMA) designation was associated with a significant decline in perinatal mortality but no significant changes in severe maternal morbidity or preterm birth or low birth weight.

A 0.39 percentage point decline (95% CI, −0.77 to −0.01 percentage points; *P* = .04; −39.5% change) in the fraction of patients who experienced a perinatal mortality event was seen at the AMA cutoff (Figure 2; Table 2). However, there were no significant changes in the proportion of individuals with severe maternal morbidity or with preterm birth or low birth weight at age 35 years.

**Table 2.** Local Linear Regression Estimates of Association Between Advanced Maternal Age, Prenatal Care Services, and Maternal and Perinatal Outcomes

Outcomes	Full sample (N = 51 290)^a^			Subgroup with low-risk pregnancy (n = 40 472)^b^		
	Age <35 y	Coefficient (95% CI)^c^	*P* value	Age <35 y	Coefficient (95% CI)	*P* value
Prenatal care services						
Total OBGYN visits, mean (SD)	8.44 (6.25)	0.23 (−0.02 to 0.47)	.07	7.54 (5.46)	0.27 (0.02 to 0.53)	.04
Any MFM visit, %	49.8	4.27 (2.27 to 6.26)	<.001	45.4	5.17 (2.91 to 7.44)	<.001
Total ultrasound scans, mean (SD)	5.45 (3.88)	0.21 (0.06 to 0.37)	.006	4.77 (3.17)	0.24 (0.08 to 0.39)	.002
Any detailed ultrasound scan, %	43.2	15.67 (13.68 to 17.66)	<.001	39.0	16.02 (13.75 to 18.28)	<.001
Any antepartum surveillance, %	51.9	4.86 (2.83 to 6.89)	<.001	46.2	5.88 (3.53 to 8.23)	<.001
Nonstress test	40.8	3.37 (1.34 to 5.40)	.001	35.8	3.85 (1.56 to 6.14)	.001
Biophysical profile	30.0	3.70 (1.88 to 5.53)	<.001	24.9	4.43 (2.44 to 6.43)	<.001
Any aneuploidy screening, %	74.5	0.99 (−0.78 to 2.76)	.27	73.8	0.59 (−1.43 to 2.60)	.57
Serum analyte	67.3	−4.02 (−5.94 to −2.09)	<.001	66.7	−4.59 (−6.77 to −2.41)	<.001
Cell-free DNA test	14.8	9.40 (7.97 to10.83)	<.001	14.4	8.71 (7.12 to 10.29)	<.001
Invasive genetic test	2.7	1.10 (0.35 to 1.85)	.004	2.5	0.70 (−0.11 to 1.52)	.09
Maternal and newborn outcomes, %						
Any severe maternal morbidity	3.5	−0.30 (−1.07 to 0.47)	.45	2.8	−0.28 (−1.06 to 0.51)	.49
Perinatal mortality	0.9	−0.39 (−0.77 to −0.01)	.04	0.4	−0.40 (−0.66 to −0.15)	.002
Preterm birth or low birth weight	12.9	0.42 (−0.98 to 1.81)	.56	9.5	0.45 (−0.98 to 1.89)	.53
Preterm birth	12.0	0.59 (−0.76 to 1.95)	.39	8.8	0.59 (−0.79 to 1.97)	.40
Low birth weight	5.9	0.29 (−0.70 to 1.28)	.56	4.1	−0.14 (−1.11 to 0.83)	.77

Abbreviations: MFM, maternal-fetal medicine; OBGYN, obstetrician-gynecologist.

^a^ The full sample includes all individuals with a delivery during the study period (2008-2019) who turned age 35 years within 120 days of the expected date of delivery. The expected date of delivery (assuming 40 weeks’ gestation) was defined based on the actual date of delivery and gestational age at delivery. Individuals were required to have continuous eligibility during entire pregnancy period, a zip code of residence in the data, and also at least 1 outpatient visit and 1 ultrasound scan during pregnancy. Individuals who turned age 35 years within 7 days of the expected date of delivery were excluded because gestational age is only measured in weeks in the data.

^b^ The subgroup of low-risk pregnancies included all mothers without a diagnosis code for pregestational diabetes, chronic hypertension, obesity, or multiple gestation before or during pregnancy.

^c^ All adjusted regressions for the full sample controlled for individual-level characteristics (pregestational diabetes, chronic hypertension, obesity, and multiple gestation) and zip code characteristics (percent White individuals, percent Hispanic individuals, median household income, and whether the zip code was urban), and county-level characteristics (any hospital with neonatal intensive care unit and OBGYNs per 10 000 deliveries). All adjusted regressions for the subgroup with a low-risk pregnancy controlled for all zip code and county-level characteristics. All adjusted regressions also included state, year, and month of delivery fixed effects.

### Subgroup Analysis, Low-risk Pregnancies

In the sample of low-risk pregnancies (n = 40 472) (eTable 5 in the Supplement), prenatal care services increased substantially at the 35-year cutoff, and in all cases, the increases at age 35 years for this group were larger than for the full sample (Table 2; eFigure 2 in the Supplement). The decline in perinatal mortality at the AMA cutoff was also substantially larger than the full sample, with a 0.40 percentage point decline (95% CI, −0.66 to −0.15 percentage points; *P* = .002; −88.2% change) (eFigure 3 in the Supplement).

### Sensitivity Analyses

No discontinuity in the number of deliveries at age 35 years was found (eFigure 4 in the Supplement), and the AMA designation was not associated with any changes in maternal, infant, or zip code or county of residence characteristics (eTable 6 in the Supplement) or the proportion of pregnancies resulting in termination or miscarriage (eTable 7 in the Supplement). Results were consistent using 90- or 150-day bandwidths (eTables 8-9 in the Supplement) and without covariate adjustment (eTable 10 in the Supplement). Only 8 of 128 placebo regressions (6.3%) were significant, suggesting that the increases in services and declines in mortality were unique to the 35-year cutoff (eTables 11 and 12 in the Supplement). A 50.44 percentage point increase (95% CI, 48.69-52.18 percentage points; *P* < .001) in the proportion of individuals with a diagnosis code for elderly primigravida or multigravida was estimated at the AMA cutoff (eTable 13, eFigure 5 in the Supplement).

## Discussion

Using a national sample of over 50 000 commercially insured individuals with deliveries between 2008 and 2019, we found that patients with an expected date of delivery just after they reached AMA were substantially less likely to experience a perinatal death than those whose expected date of delivery was just before their 35th birthday. Pronounced increases in prenatal care services were observed across the AMA cutoff, with the biggest increases seen in antepartum surveillance and detailed ultrasound scans, as well as increases in visits with MFM specialists and total ultrasound scans performed. Using a novel RD approach, we found suggestive evidence that more intensive prenatal monitoring was associated with meaningful improvements in perinatal survival in this age range.

Results from this study suggest that changes in clinical behavior stemming from the AMA designation were associated with substantial improvements in perinatal survival. Unfortunately, we were not able to determine precisely which aspects of prenatal care were associated with improvements in perinatal mortality for several reasons. First, we found several changes in clinician behavior at the 35-year cutoff; therefore, it was difficult to disentangle the individual contribution of any particular service. Second, many aspects of clinical care and decision-making cannot be captured in claims data. It is possible that important changes in clinical decision-making associated with the AMA designation (eg, interpretation of test results and timing of tests and visits) were not captured here.

The prenatal services used to monitor fetal growth and improve perinatal survival, including detailed ultrasound scans and antepartum surveillance, were the prenatal services with the largest increases at the AMA cutoff. These results are consistent with prior research, which found that pregnant individuals older than 35 years who underwent weekly antepartum surveillance had rates of stillbirth comparable to those of much younger patients.^34^ The potential for antepartum surveillance to reduce perinatal mortality is also supported by multiple observational studies, which have demonstrated its role in identifying fetuses at increased risk for stillbirth.^35,36,37,38^ However, it is important to note that most patients in our commercially insured sample were in urban, higher-income areas. Further research is needed to understand the potential for these services to reduce the high rates of perinatal mortality observed in rural areas and among minority patients.^39^

The sharp increase in prenatal care services at the AMA cutoff is an important and novel finding on its own. Prior research examining the rates of prenatal care and poor pregnancy outcomes have found increases in prenatal care visits and poor outcomes with maternal age but only examined across 5-year age bands; those findings may therefore be confounded by increases in underlying risk.^10,40,41,42^ The marked increases in prenatal services at age 35 years suggest that clinicians use the cutoff as a heuristic in their clinical recommendations and service provision. No clinical obstetric guidelines, including those from the American College of Obstetricians and Gynecologists and the Society for Maternal-Fetal Medicine, recommend using AMA alone as a determining factor in clinical decision-making for the use of services such as antepartum surveillance.^11,17^ The fact that these services increase more at the cutoff for patients with an otherwise low-risk pregnancy is supportive of the idea that clinicians use the 35-year cutoff as a factor in risk assessment and allocating care.

Our results suggest that 3.9 perinatal deaths per 1000 deliveries in this age range could be averted if patients just a few months younger than the AMA cutoff received similar care to those older than the cutoff. More rigorous evidence is needed on the value and effect of prenatal care guidelines and services on pregnancy outcomes. Better evidence on the causal effect of prenatal services is an important step in addressing the extremely inequitable incidence of maternal and perinatal morbidity and mortality in the US.^7^

High and rising rates of severe maternal morbidity in the US are particularly concerning for individuals of AMA who are at a greater risk of poor outcomes.^43^ We did not find that the AMA designation was associated with reductions in severe maternal morbidity, despite increases in prenatal care services. One explanation for this could be that increased intervention during pregnancy is too late to address the most substantial risk factors for poor outcomes, including obesity and prepregnancy hypertension and diabetes.^44,45,46^ The AMA cutoff may also influence clinician decision-making regarding the fetus more than the pregnant individual, and additional intervention during pregnancy and delivery may introduce additional risks for pregnant individuals.^47^ In addition, changes in clinician behavior at the AMA cutoff are unlikely to affect the system-level factors associated with maternal morbidity.^48^ Furthermore, with an increased risk of severe maternal morbidity after delivery among individuals of increasing maternal age, additional postpartum care may be important to reducing the high rates of morbidity.^49^ Additional research is needed to understand what aspects of preconception, prenatal, and postpartum care can reduce the high rates of severe maternal morbidity among individuals of AMA.

### Limitations

This study had several limitations. First, claims data for maternity services may not have included all relevant visits and services because a global billing code and single payment is often used for prenatal, intrapartum, and postpartum care. However, genetic tests and nonroutine services, including antepartum fetal surveillance, are often billed separately and observable in claims. With an average of 16.8 outpatient encounters and 8.4 OBGYN visits during pregnancy in our study population, it seems unlikely that we missed many visits. Second, we were unable to explore changes in delivery-related procedures, such as labor induction and planned cesarean delivery, due to underreporting in claims data and changes in coding after the switch to the *International Statistical Classification of Diseases and Related Health Problems, Tenth Revision* during our study period (eFigure 6 in the Supplement). Third, our findings may not be generalizable to other insurance types. Fourth, we lacked information on the causes of perinatal deaths and cannot explore which cases were avertable. Fifth, structural racism and systemic access barriers are important factors in pregnancy outcomes, and our data did not allow us to examine the extent to which these factors were exacerbated or mitigated by changes in clinical behavior at the 35-year cutoff. Finally, results may not be generalizable to individuals far from the AMA cutoff who may differ markedly in terms of chronic conditions and other pregnancy risk factors.

## Conclusions

Perinatal and maternal outcomes remain unacceptably poor and inequitable in the US despite increasing prenatal care use and spending. Experts have called for a fresh look at the evidence base for commonly used prenatal care guidelines and a deeper investigation into what aspects of care can improve outcomes.^7,8,10,24,50^ In this cross-sectional RD study, we found that the 35-year AMA cutoff in prenatal care was associated with substantial increases in prenatal care intensity and large declines in perinatal mortality. Increases in fetal monitoring, which aim to increase fetal survival, were largest at the cutoff, but this study cannot identify which aspects of prenatal care were most important for perinatal mortality. Additional attention is needed to understand how prenatal monitoring can be targeted toward patients who are most likely to benefit.

## Notes

Supplement. **eTable 1.** Diagnosis and Procedure Codes for Identification of Deliveries **eTable 2.** Diagnosis Codes to Identify Gestational Age at Delivery **eTable 3.** Diagnosis and Procedure Codes for Identification of Maternal Characteristics and Outcomes **eTable 4.** Sample Selection **eFigure 1.** Regression Plots for Aneuploidy Screening by Type of Screening **eTable 5.** Characteristics of Women With a Low-Risk Pregnancy Who Were Within 120 Days of Age 35 on the Expected Date of Delivery **eFigure 2.** Prenatal Care Services by Weeks Relative to Age 35 on Expected Date of Delivery Among Women With a Low-Risk Pregnancy **eFigure 3.** Perinatal and Maternal Outcomes by Weeks Relative to Age 35 on Expected Date of Delivery Among Women With a Low-Risk Pregnancy **eFigure 4.** Histogram of All Deliveries to Individuals Within 120 Days of the AMA Cutoff **eTable 6.** Test for Changes in Sample Characteristics at Age 35 Cutoff **eTable 7.** Regression Results for Changes in Termination or Miscarriage at Age 35 Cutoff **eTable 8.** Regression Results With Varying Bandwidth for All Individuals **eTable 9.** Regression Results With Varying Bandwidth for Individuals with Low-Risk Pregnancy **eTable 10.** Unadjusted Regression Results **eTable 11.** Placebo Regression Results Below Age 35 **eTable 12.** Placebo Regression Results Above Age 35 **eTable 13.** Regression Results for Diagnosis Code for Elderly Primigravida and/or Multigravida During Pregnancy **eFigure 5.** Regression Plots for Diagnosis Code for Elderly Primigravida and/or Multigravida During Pregnancy **eFigure 6.** Changes in Rate of Induction of Labor During the Study Period

## References

[1] Hoyert DL, Miniño AM. *National Vital Statistics Reports Maternal Mortality in the United States: Changes in Coding, Publication, and Data Release, 2018.* US Dept of Health and Human Services; 2020. 32510319

[2] Centers for Disease Control and Prevention. Reproductive health severe maternal morbidity. Accessed February 7, 2020. https://www.cdc.gov/reproductivehealth/maternalinfanthealth/severematernalmorbidity.html

[3] Hoyert DL. *Maternal Mortality Rates in the United States, 2019*. National Center for Health Statistics: Health E-Stats; 2021.

[4] Kassebaum NJ, Barber RM, Dandona L, ; GBD 2015 Maternal Mortality Collaborators. Global, regional, and national levels of maternal mortality, 1990-2015: a systematic analysis for the Global Burden of Disease Study 2015. Lancet. 2016;388(10053):1775-1812. doi:10.1016/S0140-6736(16)31470-2 27733286 PMC5224694

[5] Blencowe H, Cousens S, Jassir FB, ; Lancet Stillbirth Epidemiology Investigator Group. National, regional, and worldwide estimates of stillbirth rates in 2015, with trends from 2000: a systematic analysis. Lancet Glob Health. 2016;4(2):e98-e108. doi:10.1016/S2214-109X(15)00275-2 26795602

[6] Office of Disease Prevention and Health Promotion. Pregnancy and childbirth. Accessed July 9, 2021. https://health.gov/healthypeople/objectives-and-data/browse-objectives/pregnancy-and-childbirth

[7] Gourevitch R, Friedman Peahl A, McConnell M, Shah N. *Understanding The Impact Of Prenatal Care: Improving Metrics, Data, and Evaluation.* Health Affairs Blog; 2020.

[8] Peahl AF, Howell JD. The evolution of prenatal care delivery guidelines in the United States. Am J Obstet Gynecol. 2021;224(4):339-347. doi:10.1016/j.ajog.2020.12.016 33316276 PMC9745905

[9] Kilpatrick SJ, Papile L-A, Macones GA, Watterberg KL; AAP Committee on Fetus and Newborn and ACOG Committee on Obstetric Practice. *Guidelines for Perinatal Care.* 8th ed. American Academy of Pediatrics; 2017.

[10] Osterman MJK, Martin JA. Timing and adequacy of prenatal care in the United States, 2016. Natl Vital Stat Rep. 2018;67(3):1-14. 29874159

[11] American College of Obstetricians and Gynecologists. Practice bulletin no. 145: antepartum fetal surveillance. Obstet Gynecol. 2014;124(1):182-192. doi:10.1097/01.AOG.0000451759.90082.7b 24945455

[12] American College of Obstetricians and Gynecologists. Levels of maternal care: obstetric care consensus No. 9. Obstet Gynecol. 2019;134(2):e41-e55. doi:10.1097/AOG.0000000000003383 31348224

[13] American College of Obstetricians and Gynecologists Committee on Genetics. Committee Opinion No. 545: noninvasive prenatal testing for fetal aneuploidy. Obstet Gynecol. 2012;120(6):1532-1534. doi:10.1097/01.AOG.0000423819.85283.f4 23168792

[14] Peahl AF, Gourevitch RA, Luo EM, . Right-sizing prenatal care to meet patients’ needs and improve maternity care value. Obstet Gynecol. 2020;135(5):1027-1037. doi:10.1097/AOG.0000000000003820 32282594

[15] Frick KD, Lantz PM. Selection bias in prenatal care utilization: an interdisciplinary framework and review of the literature. Med Care Res Rev. 1996;53(4):371-396. doi:10.1177/107755879605300401 10162957

[16] Vintzileos AM, Ananth CV, Smulian JC, Scorza WE, Knuppel RA. Prenatal care and black-white fetal death disparity in the United States: heterogeneity by high-risk conditions. Obstet Gynecol. 2002;99(3):483-489. doi:10.1097/00006250-200203000-00019 11864678

[17] Stone J. Advanced maternal age and the risk of antepartum stillbirth. Accessed July 1, 2020. https://wftinc.org/wp-content/uploads/2019/02/SMF-Stillbirth.pdf

[18] Kenny LC, Lavender T, McNamee R, O’Neill SM, Mills T, Khashan AS. Advanced maternal age and adverse pregnancy outcome: evidence from a large contemporary cohort. PLoS One. 2013;8(2):e56583. doi:10.1371/journal.pone.0056583 23437176 PMC3577849

[19] Pritchard JA, Gant NF, MacDonald PC. Williams Obstetrics. Appleton-Century-Crofts; 1985.

[20] Khalil A, Syngelaki A, Maiz N, Zinevich Y, Nicolaides KH. Maternal age and adverse pregnancy outcome: a cohort study. Ultrasound Obstet Gynecol. 2013;42(6):634-643. doi:10.1002/uog.12494 23630102

[21] Lean SC, Derricott H, Jones RL, Heazell AEP. Advanced maternal age and adverse pregnancy outcomes: a systematic review and meta-analysis. PLoS One. 2017;12(10):e0186287. doi:10.1371/journal.pone.0186287 29040334 PMC5645107

[22] United Healthcare. Cell-free fetal DNA testing. October 1, 2021. Accessed February 23, 2021. https://www.uhcprovider.com/content/dam/provider/docs/public/policies/comm-medical-drug/cell-free-fetal-dna-testing.pdf

[23] Macdorman MF, Gregory ECW. Fetal and Perinatal Mortality: United States, 2013. US Dept of Health and Human Services; 2013. 26222771

[24] Gregory ECW, Drake P, Martin JA. Lack of change in perinatal mortality in the United States, 2014-2016. NCHS Data Brief. 2018;(316):1-8. 30089086

[25] Centers for Disease Control and Prevention. How does CDC identify severe maternal morbidity? Accessed July 1, 2020. https://www.cdc.gov/reproductivehealth/maternalinfanthealth/smm/severe-morbidity-ICD.htm

[26] Imbens G, Lemieux T. The regression discontinuity design—theory and applications. J Econom. 2008;142(2):611-614.

[27] Venkataramani AS, Bor J, Jena AB. Regression discontinuity designs in healthcare research. BMJ. 2016;352:i1216. doi:10.1136/bmj.i1216 26977086 PMC6884311

[28] Goulden R, Rowe BH, Abrahamowicz M, Strumpf E, Tamblyn R. Association of intravenous radiocontrast with kidney function: a regression discontinuity analysis. JAMA Intern Med. 2021;181(6):767-774. doi:10.1001/jamainternmed.2021.0916 33818606 PMC8022267

[29] Wallace J, Song Z. Traditional Medicare versus private insurance: how spending, volume, and price change at age sixty-five. Health Aff (Millwood). 2016;35(5):864-872. doi:10.1377/hlthaff.2015.1195 27140993 PMC4943661

[30] Cattaneo MD, Idrobo N, Titiunik R. *A Practical Introduction to Regression Discontinuity Designs*. Cambridge University Press; 2019. doi:10.1017/9781108684606

[31] Barreca AI, Guldi M, Lindo JM, Waddell GR. Saving babies? revisiting the effect of very low birth weight classification. Q J Econ. 2011;126(4):2117-1223. doi:10.1093/qje/qjr042 22256343

[32] Aetna. Antepartum fetal surveillance. Accessed February 23, 2021. http://www.aetna.com/cpb/medical/data/1_99/0088.html

[33] McCrary J. Manipulation of the running variable in the regression discontinuity design: a density test. J Econom. 2008;142(2):698-714. doi:10.1016/j.jeconom.2007.05.005

[34] Fox NS, Rebarber A, Silverstein M, Roman AS, Klauser CK, Saltzman DH. The effectiveness of antepartum surveillance in reducing the risk of stillbirth in patients with advanced maternal age. Eur J Obstet Gynecol Reprod Biol. 2013;170(2):387-390. doi:10.1016/j.ejogrb.2013.07.035 23932303

[35] Nageotte MP, Towers CV, Asrat T, Freeman RK. Perinatal outcome with the modified biophysical profile. Am J Obstet Gynecol. 1994;170(6):1672-1676. doi:10.1016/S0002-9378(94)70339-6 8203424

[36] Baskett TF, Allen AC, Gray JH, Young DC, Young LM. Fetal biophysical profile and perinatal death. Obstet Gynecol. 1987;70(3 pt 1):357-360. doi:10.1097/00132582-198804000-00004 3306498

[37] Clark SL, Sabey P, Jolley K. Nonstress testing with acoustic stimulation and amniotic fluid volume assessment: 5973 tests without unexpected fetal death. Am J Obstet Gynecol. 1989;160(3):694-697. doi:10.1016/S0002-9378(89)80062-6 2929695

[38] Miller DA, Rabello YA, Paul RH. The modified biophysical profile: antepartum testing in the 1990s. Am J Obstet Gynecol. 1996;174(3):812-817. doi:10.1016/S0002-9378(96)70305-8 8633648

[39] Healthy People.gov. Maternal, infant, and child health. Accessed July 7, 2021. https://www.healthypeople.gov/2020/data-search/Search-the-Data?nid=4824

[40] Balayla J, Azoulay L, Assayag J, Benjamin A, Abenhaim HA. Effect of maternal age on the risk of stillbirth: a population-based cohort study on 37 million births in the United States. Am J Perinatol. 2011;28(8):643-650. doi:10.1055/s-0031-1276739 21544772

[41] Reddy UM, Ko CW, Willinger M. Maternal age and the risk of stillbirth throughout pregnancy in the United States. Am J Obstet Gynecol. 2006;195(3):764-770. doi:10.1016/j.ajog.2006.06.019 16949411

[42] Cavazos-Rehg PA, Krauss MJ, Spitznagel EL, . Maternal age and risk of labor and delivery complications. Matern Child Health J. 2015;19(6):1202-1211. doi:10.1007/s10995-014-1624-7 25366100 PMC4418963

[43] Lisonkova S, Potts J, Muraca GM, . Maternal age and severe maternal morbidity: a population-based retrospective cohort study. PLoS Med. 2017;14(5):e1002307. doi:10.1371/journal.pmed.1002307 28558024 PMC5448726

[44] Bartha JL, Martinez-Del-Fresno P, Comino-Delgado R. Early diagnosis of gestational diabetes mellitus and prevention of diabetes-related complications. Eur J Obstet Gynecol Reprod Biol. 2003;109(1):41-44. doi:10.1016/S0301-2115(02)00480-3 12818441

[45] American College of Obstetricians and Gynecologists. Preeclampsia and high blood pressure during pregnancy. Accessed July 24, 2020. https://www.acog.org/patient-resources/faqs/pregnancy/preeclampsia-and-high-blood-pressure-during-pregnancy

[46] American College of Obstetricians and Gynecologists. ACOG Practice bulletin No. 190: gestational diabetes mellitus. Obstet Gynecol. 2018;131(2):e49-e64. doi:10.1097/AOG.0000000000002501 29370047

[47] Miller S, Abalos E, Chamillard M, . Beyond too little, too late and too much, too soon: a pathway towards evidence-based, respectful maternity care worldwide. Lancet. 2016;388(10056):2176-2192. doi:10.1016/S0140-6736(16)31472-6 27642019

[48] Collier AY, Molina RL. Maternal mortality in the United States: updates on trends, causes, and solutions. Neoreviews. 2019;20(10):e561-e574. doi:10.1542/neo.20-10-e561 31575778 PMC7377107

[49] Chen J, Cox S, Kuklina EV, Ferre C, Barfield W, Li R. Assessment of incidence and factors associated with severe maternal morbidity after delivery discharge among women in the US. JAMA Netw Open. 2021;4(2):e2036148-e2036148. doi:10.1001/jamanetworkopen.2020.36148 33528553 PMC7856547

[50] Hirshberg A, Srinivas SK. Epidemiology of maternal morbidity and mortality. Semin Perinatol. 2017;41(6):332-337. doi:10.1053/j.semperi.2017.07.007 28823579

