# Supplementary material for: Association of Prenatal Care Services, Maternal Morbidity, and Perinatal Mortality With the Advanced Maternal Age Cutoff of 35 Years
Source: JAMA Health Forum. 2021 Dec 3;2(12):e214044. doi: 10.1001/jamahealthforum.2021.4044 (PMC8796879; doi:10.1001/jamahealthforum.2021.4044)
Supplement: Supplement. — eTable 1. Diagnosis and Procedure Codes for Identification of Deliveries eTable 2. Diagnosis Codes to Identify Gestational Age at Delivery eTable 3. Diagnosis and Procedure Codes for Identification of Maternal Characteristics and Outcomes eTable 4. Sample Selection eFigure 1. Regression Plots for Aneuploidy Screening by Type of Screening eTable 5. Characteristics of Women With a Low-Risk Pregnancy Who Were Within 120 Days of Age 35 on the Expected Date of Delivery eFigure 2. Prenatal Care Services by Weeks Relative to Age 35 on Expected Date of Delivery Among Women With a Low-Risk Pregnancy eFigure 3. Perinatal and Maternal Outcomes by Weeks Relative to Age 35 on Expected Date of Delivery Among Women With a Low-Risk Pregnancy eFigure 4. Histogram of All Deliveries to Individuals Within 120 Days of the AMA Cutoff eTable 6. Test for Changes in Sample Characteristics at Age 35 Cutoff eTable 7. Regression Results for Changes in Termination or Miscarriage at Age 35 Cutoff eTable 8. Regression Results With Varying Bandwidth for All Individuals eTable 9. Regression Results With Varying Bandwidth for Individuals with Low-Risk Pregnancy eTable 10. Unadjusted Regression Results eTable 11. Placebo Regression Results Below Age 35 eTable 12. Placebo Regression Results Above Age 35 eTable 13. Regression Results for Diagnosis Code for Elderly Primigravida and/or Multigravida During Pregnancy eFigure 5. Regression Plots for Diagnosis Code for Elderly Primigravida and/or Multigravida During Pregnancy eFigure 6. Changes in Rate of Induction of Labor During the Study Period [file jamahealthforum-e214044-s001.pdf]

Supplementary Online Content

Geiger CK, Clapp MA, Cohen JL. Association of prenatal care services, maternal morbidity, and perinatal mortality with the advanced maternal age cutoff of 35 years. *JAMA Health Forum*. 2021;2(12):e214044. doi:10.1001/jamahealthforum.2021.4044

eTable 1. Diagnosis and Procedure Codes for Identification of Deliveries

eTable 2. Diagnosis Codes to Identify Gestational Age at Delivery

eTable 3. Diagnosis and Procedure Codes for Identification of Maternal Characteristics and Outcomes

eTable 4. Sample Selection

eFigure 1. Regression Plots for Aneuploidy Screening by Type of Screening

eTable 5. Characteristics of Women With a Low-Risk Pregnancy Who Were Within 120 Days of Age 35 on the Expected Date of Delivery

eFigure 2. Prenatal Care Services by Weeks Relative to Age 35 on Expected Date of Delivery Among Women With a Low-Risk Pregnancy

eFigure 3. Perinatal and Maternal Outcomes by Weeks Relative to Age 35 on Expected Date of Delivery Among Women With a Low-Risk Pregnancy

eFigure 4. Histogram of All Deliveries to Individuals Within 120 Days of the AMA Cutoff

eTable 6. Test for Changes in Sample Characteristics at Age 35 Cutoff

eTable 7. Regression Results for Changes in Termination or Miscarriage at Age 35 Cutoff

eTable 8. Regression Results With Varying Bandwidth for All Individuals

eTable 9. Regression Results With Varying Bandwidth for Individuals with Low-Risk Pregnancy

eTable 10. Unadjusted Regression Results

eTable 11. Placebo Regression Results Below Age 35

eTable 12. Placebo Regression Results Above Age 35

© 2021 Geiger CK et al. *JAMA Health Forum.*

eTable 13. Regression Results for Diagnosis Code for Elderly Primigravida and/or Multigravida During Pregnancy

eFigure 5. Diagnosis Codes for Elderly Primigravida and/or Multigravida During Pregnancy by Weeks Relative to Age 35 on Expected Date of Delivery

eFigure 6. Changes in Rate of Induction of Labor During the Study Period

This supplementary material has been provided by the authors to give readers additional information about their work

© 2021 Geiger CK et al. *JAMA Health Forum.*

eTable 1. Diagnosis and Procedure Codes for Identification of Deliveries

Code Type	Codes
**ICD-9 Diagnosis Codes**	V27, V30-V39, 669.71, 649.81, 649.82, 650, 669.61, 669.51, 669.01, 669.02, 669.11, 669.12, 669.81, 669.82, 669.81, 669.82
**ICD-10 Diagnosis Codes**	Z37, Z38, O75.82, O82, O90.0, P03.4, O80, O66.41, O86.13, O30.009, O75.0, O75.2, O75.5, O75.8, O75.9 O34.21
**ICD-9 Procedure Codes**	72, 73, 74
**ICD-10 Procedure Codes**	10D00Z0, 10D00Z1, 10D00Z2, 10D07Z3, 10D07Z4, 10D07Z5, 10D07Z6, 10D07Z7, 10D07Z8, 10E0XZZ
**CPT Codes**	59400, 59409, 59410, 59510, 59514, 59515, 59525, 59605, 59611, 59612, 59614, 59618, 59620, 59622, 59610, 01960, 01961, 01967, 01968, 01969
**DRG**	765, 766, 767, 768, 774, 775, 783, 784, 785, 786, 787, 788, 796, 797, 798, 805, 806, 807

Deliveries were identified in the data using a combination of procedure and diagnosis codes, detailed in eTable 1. Individuals were required to have at least one delivery-related diagnosis and procedure code on the date of delivery. Infants were linked to individuals' deliveries based on a shared subscriber identification number and an infant was only linked if the individual had an identified delivery date within seven days of the infant's recorded date of birth.

© 2021 Geiger CK et al. *JAMA Health Forum.*

eTable 2. Diagnosis Codes to Identify Gestational Age at Delivery

**Gestational Age**	**ICD-9**	**ICD-10**
**Weeks**		
≤ 7		Z3A.01
8		Z3A.08
9		Z3A.09
10		Z3A.10
11		Z3A.11
12		Z3A.12
13		Z3A.13
14		Z3A.14
15		Z3A.15
16		Z3A.16
17		Z3A.17
18		Z3A.18
19		Z3A.19
20		Z3A.20
21		Z3A.21
22		Z3A.22
23	765.21	Z3A.23, P07.22
24		Z3A.24, P07.23
25		Z3A.25, P07.24
26	765.23	Z3A.26, P07.25
27		Z3A.27, P07.26
28	765.24	Z3A.28, P07.31
29		Z3A.29, P07.32
30	765.25	Z3A.30, P07.33
31		Z3A.31, P07.34
32	765.26	Z3A.32, P07.35
33		Z3A.33, P07.36
34	765.27	Z3A.34, P07.37
35		Z3A.35, P07.38
36	765.28	Z3A.36, P07.39
37		Z3A.37
38		Z3A.38, O75.82
39		Z3A.39
40		Z3A.40
41	766.21, 645.1	Z3A.41, P08.21, O48.0
42		Z3A.42
43	766.22, 645.2	Z3A.49, P08.22, O48.1
**Extremely preterm birth (< 28 weeks)**	765.0, 765.21, 765.22, 765.23	P07.2, O60.12
**Preterm birth (< 37 weeks)**	644.21, 765.0, 765.1, 765.21-765.28	P07.2, P07.3, O60.1, O60.3

© 2021 Geiger CK et al. *JAMA Health Forum.*

**Full term birth (40 weeks)**	650, 765.29	O80, O60.2
**Post term birth (> 40 weeks)**	645	P08.2

Gestational age at delivery was calculated using diagnosis codes during pregnancy and delivery. ICD codes were used to identify the number of weeks of gestation during pregnancy and gestational age at delivery was calculated based on the number of weeks between the date of service with the gestational age code and the delivery date (eTable 2). If gestational age was not specified at any visit during pregnancy, then gestational age was identified based on codes specified at delivery. If a specific week of gestation was not specified during pregnancy or delivery, then gestational age was coded as 27 weeks if there was a code for extremely preterm birth, 36 weeks if there was a code for preterm birth, 40 weeks if there was a code for full term delivery, and 41 weeks if there was a code for a post term delivery. If there were no diagnosis codes indicating gestational age, it was assumed that the delivery was full term and gestational age was coded as 40 weeks. Among all deliveries that occurred before October 1, 2016 when ICD-10 codes were implemented, 86.0% of deliveries had at least one code from eTable 2. Among deliveries that occurred on or after October 1, 2016, 98.7% of deliveries had at least one code from eTable 2.

eTable 3. Diagnosis and Procedure Codes for Identification of Maternal Characteristics and Outcomes

**Maternal Characteristics and Outcomes**	**ICD-9**	**ICD-10**	**CPT and Other Codes**
**Diagnoses**			
Pregestational diabetes	250, V58.67, 648.0	O24.0, O24.1, O24.3, O24.8, O24.9, Z79.4, Z79.84, E10, E11, E13	
Gestational diabetes[1]	648.8	O24.4, O99.81	
Chronic hypertension	642.0, 642.1, 642.2	O10	
Gestational hypertension[2]	642.3, 642.9	O13, O16	
Preeclampsia	642.4, 642.5, 642.7	O11, O14	
Eclampsia	642.6	O15	
Obesity	278.00, 278.01, 278.03, V85.3, V85.4, 649.1	E66.0, E66.1, E66.2, E66.8, E66.9, Z68.3, Z68.4, O99.21	
Multiple gestation	651, 646.0, 678.1, V27.1, V27.3, V27.4, V27.5, V27.6, V27.7, V91, V31, V32, V33, V34, V35, V36, V37	O30, O31, Z37.2, Z37.3, Z37.4, Z37.5, Z37.6, Z37.3, Z38.3, Z38.4, Z38.5, Z38.6, Z38.7, Z38.8, O3x.xx2, O3x.xx3, O3x.xx4, O3x.xx5, O40.xx2, O40.xx3, O40.xx4, O40.xx5, O41.xx2, O41.xx3, O41.xx4, O41.xx5	
Trisomy 21	758.0	Q90.9	
Elderly primigravida and/or multigravida	V23.81, V23.82, 659.50, 659.51, 659.53, 659.60, 659.61, 659.63	O09.51, O09.52	
**Prenatal Care Services**			
Obstetrician-gynecologist visits			Encounter with servicing provider specialty recorded as obstetrician-gynecologist
Maternal-fetal medicine visits			Encounter with servicing provider specialty recorded as maternal-fetal medicine specialist
Ultrasounds			76800-76819, 76820, 76821, 76825, 76826, 76827, 76828

© 2021 Geiger CK et al. *JAMA Health Forum.*

Detailed Ultrasound			76811, 76812
Antepartum surveillance			
Biophysical profile			76818, 76819
Non-stress test			59025, 76818
Aneuploidy screening			
Serum analyte			Part 1: 84163, 84702; Part 2: 82105, 82677, 84702, 86336; with ultrasound
Cell-free DNA			81507, 81420, 0168U 0009M, 0060U
Invasive testing			59000, 59001, 76946, 59015, 76945, 59012
**Perinatal Outcomes**			
Perinatal mortality			
Stillbirth (including intrauterine death), at 28 weeks gestation or later	V27.1, V27.3, V27.4, V27.6, V27.7, 656.4	Z37.1, Z37.3, Z37.4, Z37.6, Z37.7, P95, O36.4	
Neonatal death within 7 days of delivery (including death during labor)	768.0, 768.1, 798	R99	Discharge status of 'expired' in newborn's claim within 7 days of delivery
Low birth weight	764.01-764.08, 764.11-764.18, 764.21-764.28, 764.91-764.98, 765.01-765.08, 765.11-765.18, V21.3,	P07.0, P07.1, P05.01-P05.08, P05.11-P05.18	
Preterm birth (< 37 weeks)[3]	644.21, 765.0, 765.1, 765.21, 765.22, 765.23, 765.24, 765.25, 765.26, 765.27, 765.28	O60.1, P07.2, P07.3, O60.3	
**Notes:**			
1. Gestational diabetes not flagged if the individual already had a diagnosis for pregestational diabetes.			
2. Gestational hypertension not flagged if the individual already had a diagnosis for chronic hypertension.			
3. Delivery was also identified as preterm birth if gestational age at delivery was calculated to be < 37 weeks.			

© 2021 Geiger CK et al. *JAMA Health Forum.*

eTable 4. Sample Selection

	**Total Deliveries**
0. All deliveries	1,919,868
1. All deliveries with expected date of delivery within 120 days of AMA cutoff (date of 35th birthday)	75,198
2. Any eligibility data	74,946
3. Continuous eligibility during pregnancy period	56,208
4. Valid zip code	55,957
5. Outpatient visit and ultrasound during pregnancy	54,745
6. Exclude deliveries within 7 days of AMA cutoff	51,290

© 2021 Geiger CK et al. *JAMA Health Forum.*

eFigure 1. Regression Plots for Aneuploidy Screening by Type of Screening

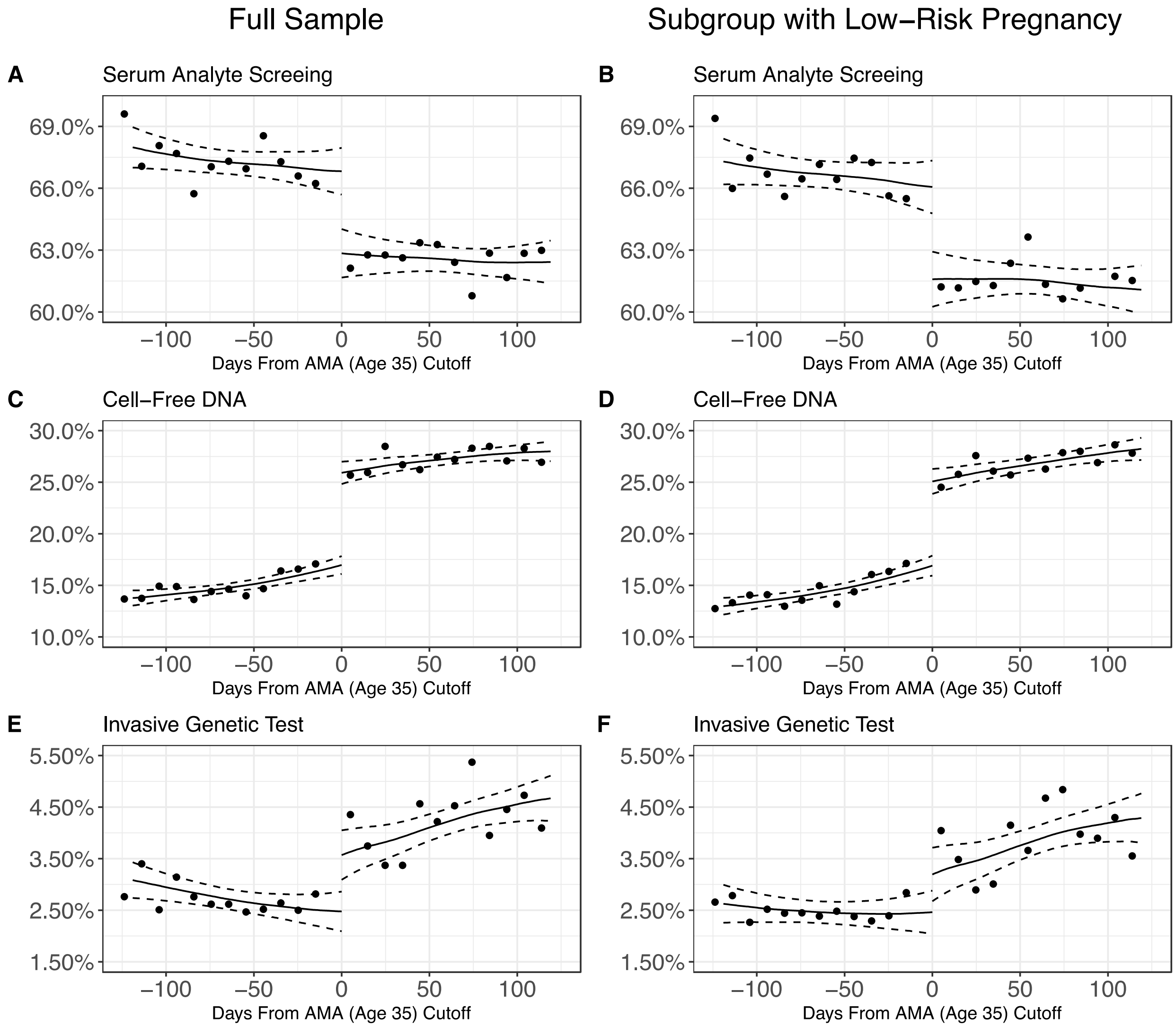

Legend: Shown are the binned unadjusted outcomes plotted by the running variable, i.e., the number of days between the expected date of delivery and 35th birthday. The solid lines represent the adjusted local linear regression results for the regression discontinuity analyses. Dotted lines represent the 95% confidence intervals. The sample included individuals with an expected date of delivery within 120 days of her 35th birthday. Women with an expected date of delivery within 7 days of the 35th birthday were excluded.

© 2021 Geiger CK et al. *JAMA Health Forum.*

eTable 5. Characteristics of Women With a Low-Risk Pregnancy Who Were Within 120 Days of Age 35 on the Expected Date of Delivery

	**Women with Low-Risk Pregnancy[1]**	
**Characteristics**	**34.7-34.9 years**	**35.0-35.3 years**
Total Deliveries, N	20,650	19,822
Prenatal Care Services		
Total OBGYN visits, mean (SD)	7.54 (5.46)	8.06 (5.75)
Any MFM visit, n (%)	9,369 (45.37%)	10,347 (52.2%)
Any aneuploidy screening, n (%)	15,243 (73.82%)	15,063 (75.99%)
Serum analyte	13,774 (66.7%)	12,184 (61.47%)
Cell-free DNA	2,966 (14.36%)	5,332 (26.9%)
Invasive test[2]	511 (2.47%)	768 (3.87%)
Total ultrasound visits, mean (SD)	4.77 (3.17)	5.17 (3.47)
Any detailed ultrasound, n (%)	8,045 (38.96%)	12,272 (61.91%)
Any antepartum fetal surveillance, n (%)	9,539 (46.19%)	10,417 (52.55%)
Fetal non-stress test	7,383 (35.75%)	8,068 (40.7%)
Biophysical profile	5,139 (24.89%)	5,854 (29.53%)
Maternal and newborn outcomes, n (%)		
Any severe maternal morbidity	580 (2.81%)	558 (2.82%)
Perinatal mortality	87 (0.42%)	68 (0.34%)
Preterm birth or low birth weight	1,970 (9.54%)	2,004 (10.11%)
Preterm birth (< 37 weeks)	1,821 (8.82%)	1,840 (9.28%)
Low birth weight (< 2500 grams)	982 (3.74%)	978 (3.99%)

Abbreviations*: OBGYN = obstetrician-gynecologist; SD = standard deviation; MFM = maternal-fetal medicine.*

Notes:
1. Sample includes all individuals with a delivery during the study period (2008-2019) who turned age 35 within 120 days of the expected date of delivery. The expected date of delivery (assuming 40 weeks gestation) was defined based on the actual date of delivery and gestational age at delivery. Women were required to have continuous eligibility during entire pregnancy period, have a non-missing zip code of residence in the data, and also have at least one outpatient visit and one ultrasound during pregnancy. Women who turned age 35 within 7 days of the expected date of delivery were excluded since gestational age is only measured in weeks in the data. Individuals with a low-risk pregnancy include all individuals without a diagnosis code for pregestational diabetes, chronic hypertension, obesity, or multiple gestation.
2. Invasive genetic testing includes amniocentesis or chorionic villus sampling

© 2021 Geiger CK et al. *JAMA Health Forum.*

eFigure 2. Prenatal Care Services by Weeks Relative to Age 35 on Expected Date of Delivery Among Women With a Low-Risk Pregnancy

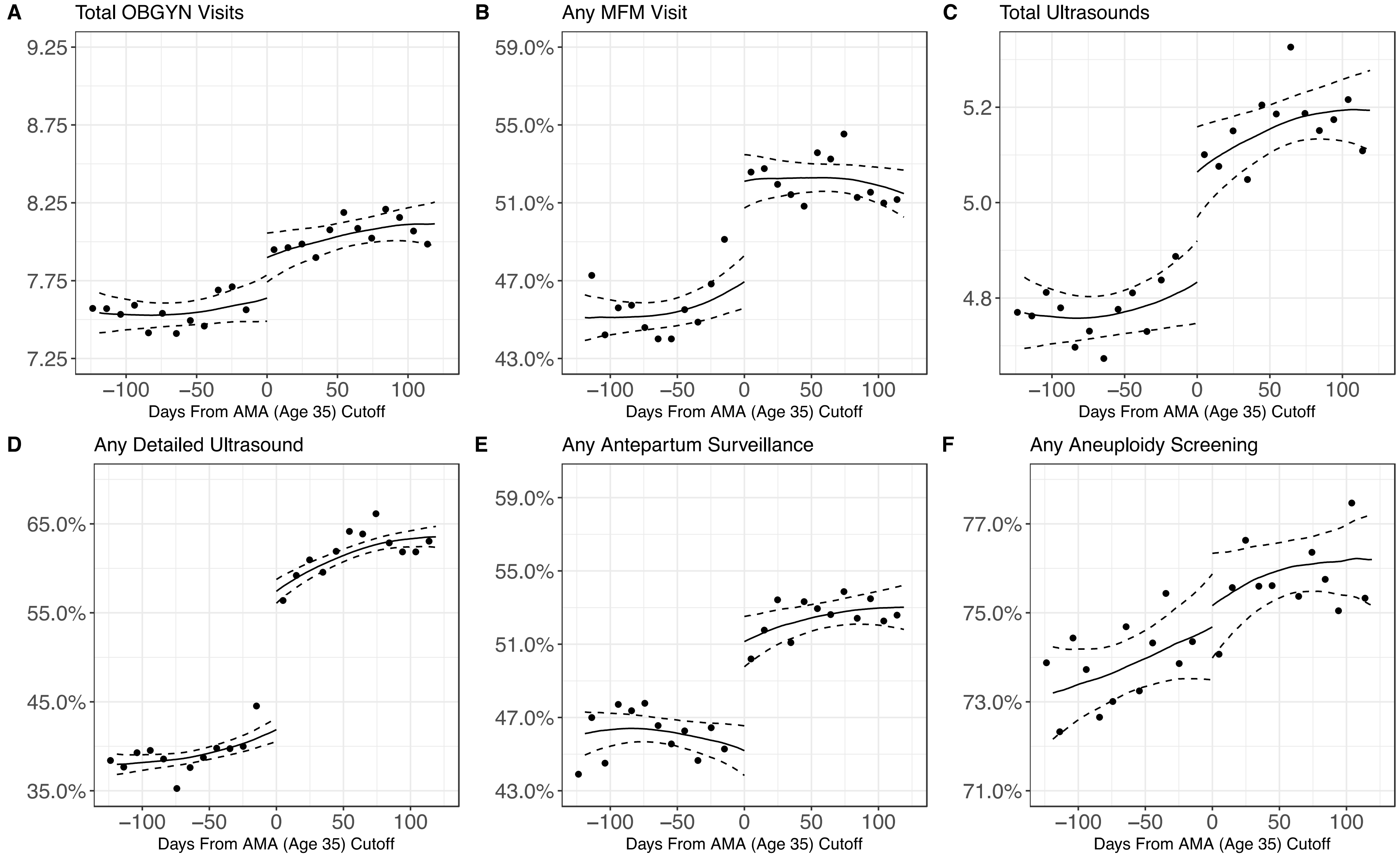

Legend: The figure shows adjusted local linear regression results for the regression discontinuity analyses (solid lines) and the 95% confidence intervals (dashed lines). The points in the figure show the binned unadjusted outcomes plotted by the running variable (i.e., the number of days between the expected date of delivery and 35th birthday). All figures show results for the subgroup with a low-risk pregnancy (N=40,472). All individuals in the sample had an expected date of delivery within 120 days of their 35th birthday. As seen in the figure and the regression results, the AMA designation was associated with significant increases in total obstetrician-gynecologist visits, any maternal-fetal medicine visits, total ultrasounds, any detailed ultrasound, and any antepartum fetal surveillance but no significant changes in total obstetrician-gynecologist visits or aneuploidy screening.

© 2021 Geiger CK et al. *JAMA Health Forum.*

eFigure 3. Perinatal and Maternal Outcomes by Weeks Relative to Age 35 on Expected Date of Delivery Among Women With a Low-Risk Pregnancy

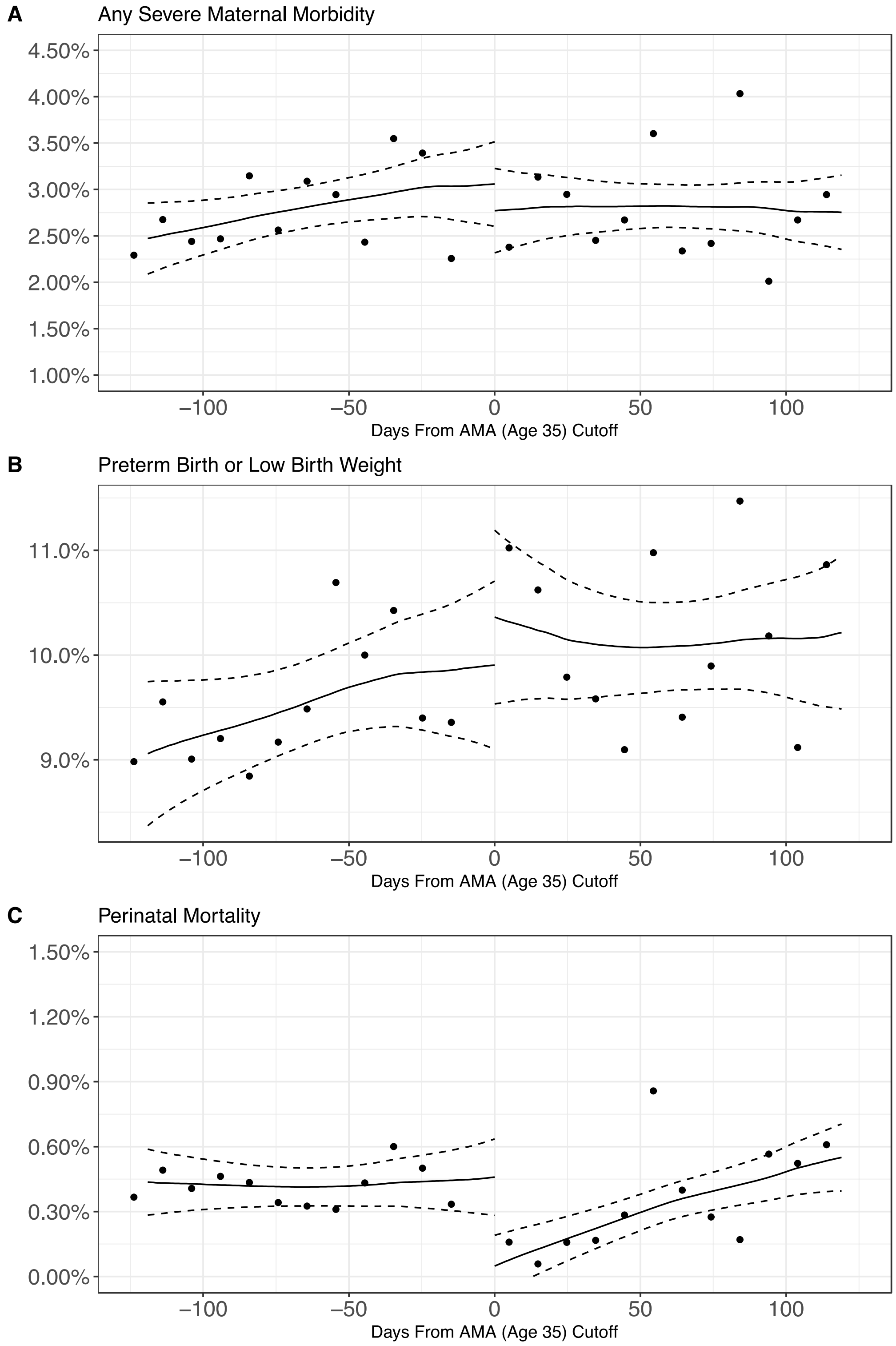

© 2021 Geiger CK et al. *JAMA Health Forum.*

Legend: The figure shows adjusted local linear regression results for the regression discontinuity analyses (solid lines) and the 95% confidence intervals (dashed lines). The points in the figure show the binned unadjusted outcomes plotted by the running variable (i.e., the number of days between the expected date of delivery and 35th birthday). All figures show results for the subgroup with a low-risk pregnancy (N=40,472). All individuals in the sample had an expected date of delivery within 120 days of their 35th birthday. As seen in the figure and the regression results, the AMA designation was associated with a significant decline in perinatal mortality but no significant changes in severe maternal morbidity or preterm birth or low birth weight.

In our study, the regression discontinuity design relies on the assumption that there is no manipulation of the running variable (i.e., days between expected date of birth and the individual's 35th birthday) so that individuals just above and below are the same on average except for the fact that women above the cutoff are of AMA. This assumption may be violated if individuals are able to manipulate the timing of their expected date of delivery to be on one side of the AMA cutoff; however, we do not expect this to occur due to the difficulty in timing an expected date of delivery. In addition, this assumption may be violated if there were differences in the outcome of pregnancies among individuals just above and below the age 35 cutoff. Due to the increase in genetic testing at age 35, it is plausible that we could see a decline in pregnancies just above the cutoff if individuals choose to terminate the pregnancy based on the results of the genetic test. To test this assumption of the regression discontinuity design, we first conduct a McCrary test which provides a formal test for manipulation of the running variable.[21] Specifically, the McCrary test formally tests whether there are any differences in the marginal density of the running variable.

Using the McCrary test, we do not find any evidence of manipulation of the running variable (p=0.249). As seen in eFigure 4, the number of deliveries appears to decline smoothly across the AMA cutoff.

© 2021 Geiger CK et al. *JAMA Health Forum.*

eFigure 4. Histogram of All Deliveries to Individuals Within 120 Days of the AMA Cutoff

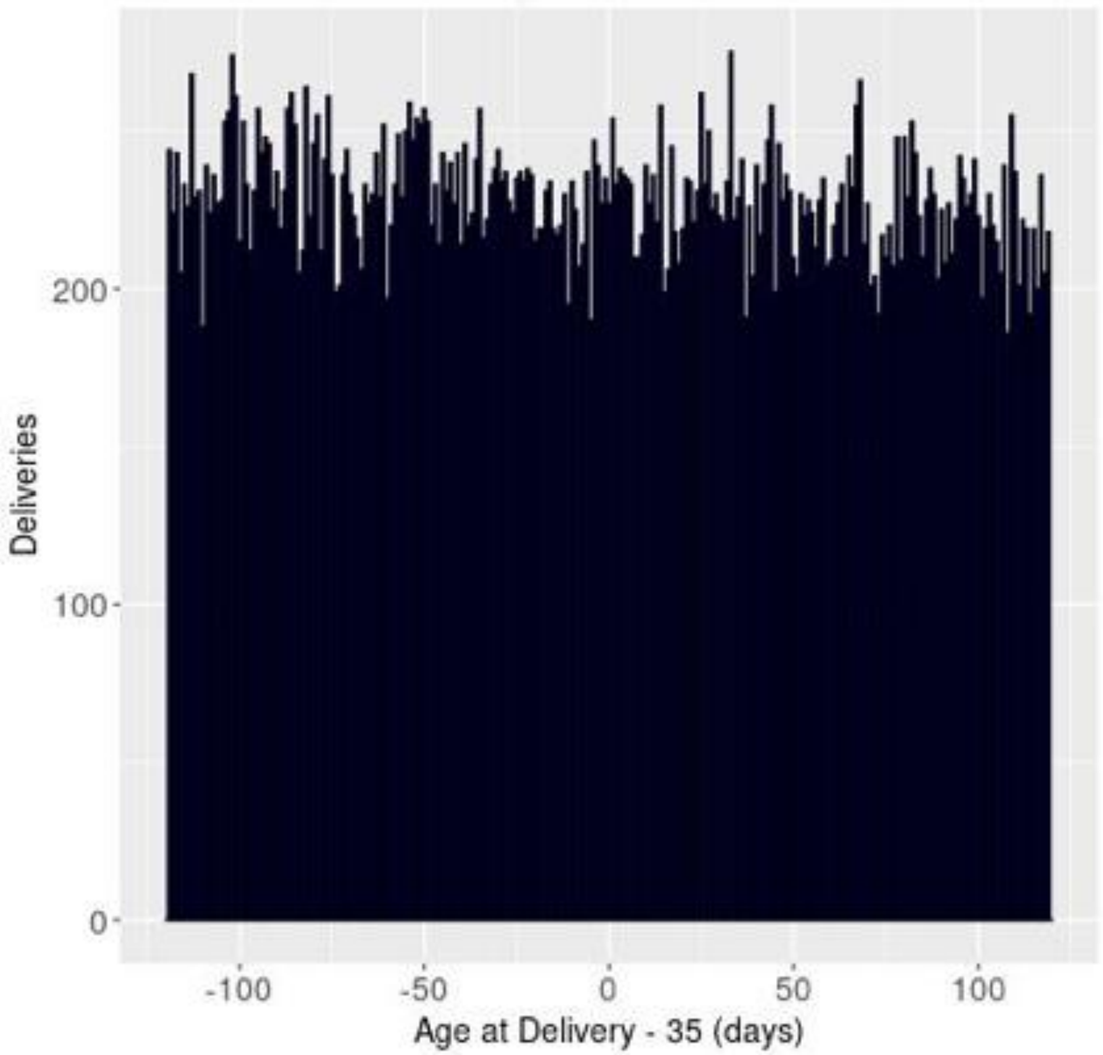

Legend: Shown is the number of deliveries by the running variable, i.e., the number of days between the expected date of delivery and the individual's 35th birthday. Sample includes all individuals with an expected date of delivery within 120 days of the 35th birthday.

© 2021 Geiger CK et al. *JAMA Health Forum.*

eTable 6. Test for Changes in Sample Characteristics at Age 35 Cutoff

	**Full Sample (N = 51,290)**	
**Outcomes**	**Coefficient (95% CI)**	**P-value**
Infant characteristics		
Trisomy 21	0.001 (0.000, 0.003)	0.107
Maternal characteristics		
Any high-risk pregnancy diagnoses	0.003 (-0.017, 0.023)	0.771
Chronic hypertension	0.003 (-0.006, 0.012)	0.469
Pregestational diabetes	-0.006 (-0.015, 0.003)	0.167
Obesity	0.002 (-0.011, 0.014)	0.809
Multiple gestation	0.003 (-0.006, 0.012)	0.469
Zip code/county of residence characteristics		
Median household income	-490.109 (-1699.641, 719.422)	0.427
% White	-0.074 (-0.774, 0.625)	0.835
% Hispanic	-0.019 (-0.581, 0.542)	0.946
Urban RUCA	0.003 (-0.012, 0.019)	0.677
Any NICU	0.001 (-0.014, 0.017)	0.891
OBGYNs per 10,000 deliveries	1.192 (-1.444, 3.828)	0.376

Abbreviations: CI = confidence interval; RUCA = rural-urban commuting areas; NICU = neonatal intensive care unit; OBGYN = obstetrician-gynecologist

Notes:

Sample includes all individuals with an expected date of delivery within 120 days of her 35th birthday. Individuals with an expected date of delivery within 7 days of the 35th birthday were excluded. All regressions for maternal and infant characteristics control for zip-code characteristics (percent white, percent Hispanic, median household income, and whether the zip code is urban) and county-level characteristics (any hospital with neonatal intensive care unit and OBGYNs per 10,000 deliveries). All regressions for zip code/county of residence control for chronic hypertension, pregestational diabetes, obesity, and multiple gestation. All regressions include state of residence, year, and month of delivery fixed effects.

© 2021 Geiger CK et al. *JAMA Health Forum.*

Pregnancy Outcomes

To test whether there were any potential changes in the sample of individuals just above or below the cutoff, we also conducted a sensitivity analysis on the outcomes of all pregnancies. Instead of limiting to only livebirths or stillborn analyses, we expanded the sample to include all pregnancies to individuals around the age 35 AMA cutoff. Then, we identified the outcome of the pregnancy based on diagnosis and procedure codes.

Using regression discontinuity methods similar to those previously described, we analyzed whether there were any changes in the outcomes of all pregnancies at the age 35 cutoff. In addition, we also analyzed changes in the outcomes of low-risk pregnancies, defined as pregnancies among individuals without a diagnosis code for pregestational diabetes, chronic hypertension, preeclampsia, eclampsia, obesity, or multiple gestation. Due to the limitations of the claims data which does not always allow for differentiation between miscarriages and pregnancy terminations, we also analyzed the proportion of pregnancies that ended in either a termination or miscarriage.

Results of the analysis are included in eTable 7. We did not find any change in the proportion of pregnancies ending in termination and/or miscarriage at the AMA cutoff. These findings provide evidence to support the assumption made in our main analyses that individuals on either side of the age 35 cutoff are the same on average and the AMA sample is unlikely to be biased due to any increase in terminations due to increases in genetic testing.

© 2021 Geiger CK et al. *JAMA Health Forum.*

eTable 7. Regression Results for Changes in Termination or Miscarriage at Age 35 Cutoff

	All Pregnancies (N = 136,477)		Low-Risk Pregnancies (N = 98,143)	
**Outcomes**	**Coefficient (95% CI)**	**P-value**	**Coefficient (95% CI)**	**P-value**
End of pregnancy				
Termination or miscarriage	-0.004 (-0.013, 0.005)	0.369	-0.006 (-0.017, 0.006)	0.332
Termination	-0.002 (-0.006, 0.002)	0.225	-0.002 (-0.008, 0.003)	0.373
Miscarriage	-0.002 (-0.010, 0.007)	0.703	-0.003 (-0.014, 0.007)	0.550
Abbreviations: CI = confidence interval.				
**Notes:**				
1. All regressions included state of residence, year, and month of delivery fixed effects.				
2. Sample limited to all pregnancies with an expected date of delivery within 120 days of the individual's 35th birthday. Pregnancies with an expected date of delivery within 7 days of the AMA cutoff were not included in the analyses.				
3. Low-risk pregnancies included all pregnancies among individuals without a diagnosis code for pregestational diabetes, chronic hypertension, obesity, or multiple gestation.				

© 2021 Geiger CK et al. *JAMA Health Forum.*

eTable 8. Regression Results With Varying Bandwidth for All Individuals

	**Full Sample - Adjusted**					
	**90 Day Bandwidth (N = 37,596)**		**120 Day Bandwidth (N = 51,290)**		**150 Day Bandwidth (N = 64,831)**	
**Outcomes**	**Coefficient (95% CI)**	**P-value**	**Coefficient (95% CI)**	**P-value**	**Coefficient (95% CI)**	**P-value**
Prenatal Care Services						
Total OBGYN visits	0.121 (-0.174, 0.416)	0.42	0.236 (-0.008, 0.480)	0.06	0.318 (0.105, 0.531)	0.003
Any MFM visit	0.030 (0.006, 0.054)	0.02	0.043 (0.023, 0.063)	<0.001	0.049 (0.032, 0.066)	<0.001
Total ultrasounds	0.157 (-0.027, 0.341)	0.10	0.222 (0.070, 0.373)	0.004	0.274 (0.143, 0.405)	<0.001
Any detailed ultrasound	0.134 (0.109, 0.158)	<0.001	0.157 (0.137, 0.177)	<0.001	0.169 (0.151, 0.186)	<0.001
Any antepartum fetal surveillance	0.045 (0.021, 0.069)	<0.001	0.050 (0.030, 0.070)	<0.001	0.052 (0.035, 0.069)	<0.001
Non-stress test	0.026 (0.001, 0.050)	0.04	0.035 (0.015, 0.055)	<0.001	0.040 (0.023, 0.057)	<0.001
Biophysical profile	0.039 (0.017, 0.061)	<0.001	0.038 (0.020, 0.056)	0.001	0.036 (0.020, 0.052)	<0.001
Any aneuploidy screening	0.003 (-0.019, 0.024)	0.80	0.010 (-0.008, 0.028)	0.26	0.014 (-0.001, 0.030)	0.06
Serum analyte	-0.043 (-0.066, -0.019)	<0.001	-0.040 (-0.059, -0.021)	<0.001	-0.039 (-0.056, -0.023)	<0.001
Cell-free DNA test	0.085 (0.067, 0.102)	<0.001	0.094 (0.080, 0.109)	<0.001	0.100 (0.088, 0.113)	<0.001
Invasive genetic test	0.010 (0.000, 0.019)	0.04	0.011 (0.004, 0.018)	0.004	0.011 (0.005, 0.018)	0.001
Maternal and Newborn Outcomes						
Any severe maternal morbidity	-0.002 (-0.012, 0.007)	0.65	-0.003 (-0.011, 0.005)	0.45	-0.003 (-0.010, 0.004)	0.42
Perinatal mortality	-0.005 (-0.009, 0.000)	0.05	-0.004 (-0.008, 0.000)	0.04	-0.003 (-0.007, 0.000)	0.04
Preterm birth or low birth weight	0.010 (-0.007, 0.027)	0.23	0.004 (-0.010, 0.018)	0.56	0.004 (-0.008, 0.016)	0.52
Preterm birth (< 37 weeks)	0.012 (-0.005, 0.028)	0.17	0.006 (-0.008, 0.019)	0.39	0.005 (-0.007, 0.017)	0.39
Low birth weight (< 2500 grams)	0.006 (-0.006, 0.018)	0.29	0.003 (-0.007, 0.013)	0.56	0.002 (-0.007, 0.011)	0.66

Abbreviations: CI = confidence interval; OBGYN = obstetrician-gynecologist; MFM = maternal-fetal medicine.

Notes:

1. Sample includes all individuals with an expected date of delivery within 120 days of her 35th birthday. Individuals with an expected date of delivery within 7 days of the 35th birthday were excluded.

2. All regressions control for individual-level characteristics (pregestational diabetes, chronic hypertension, obesity, multiple gestation), zip-code characteristics (percent white, percent Hispanic, median household income, and whether the zip code is urban), and county-level characteristics (any hospital with neonatal intensive care unit and OBGYNs per 10,000 deliveries). All regressions include state of residence, year, and month of delivery fixed effects.

© 2021 Geiger CK et al. *JAMA Health Forum.*

eTable 9. Regression Results With Varying Bandwidth for Individuals With Low-Risk Pregnancy

	**Subgroup with Low-Risk Pregnancy - Adjusted**					
	**90 Day Bandwidth (N = 29,705)**		**120 Day Bandwidth (N = 40,472)**		**150 Day Bandwidth (N = 51,578)**	
**Outcomes**	**Coefficient (95% CI)**	**P-value**	**Coefficient (95% CI)**	**P-value**	**Coefficient (95% CI)**	**P-value**
Prenatal Care Services						
Total OBGYN visits	0.164 (-0.145, 0.473)	0.30	0.273 (0.018, 0.529)	0.04	0.338 (0.115, 0.561)	0.003
Any MFM visit	0.038 (0.010, 0.065)	0.008	0.052 (0.029, 0.074)	<0.001	0.058 (0.039, 0.078)	<0.001
Total ultrasounds	0.157 (-0.029, 0.344)	0.10	0.238 (0.085, 0.391)	0.002	0.288 (0.155, 0.422)	<0.001
Any detailed ultrasound	0.135 (0.107, 0.163)	<0.001	0.160 (0.138, 0.183)	<0.001	0.175 (0.155, 0.194)	<0.001
Any antepartum fetal surveillance	0.053 (0.025, 0.082)	<0.001	0.059 (0.035, 0.082)	<0.001	0.060 (0.040, 0.080)	<0.001
Non-stress test	0.028 (0.000, 0.056)	0.05	0.039 (0.016, 0.061)	0.001	0.043 (0.023, 0.063)	<0.001
Biophysical profile	0.046 (0.022, 0.071)	<0.001	0.044 (0.024, 0.064)	<0.001	0.042 (0.024, 0.059)	<0.001
Any aneuploidy screening	-0.002 (-0.026, 0.023)	0.901	0.006 (-0.014, 0.026)	0.57	0.010 (-0.007, 0.027)	0.26
Serum analyte	-0.049 (-0.076, -0.022)	<0.001	-0.046 (-0.068, -0.024)	<0.001	-0.045 (-0.064, -0.026)	<0.001
Cell-free DNA test	0.076 (0.057, 0.096)	<0.001	0.087 (0.071, 0.103)	<0.001	0.094 (0.081, 0.108)	<0.001
Invasive genetic test	0.006 (-0.004, 0.016)	0.22	0.007 (-0.001, 0.015)	0.09	0.007 (0.000, 0.014)	0.04
Maternal and Newborn Outcomes						
Any severe maternal morbidity	-0.002 (-0.011, 0.008)	0.75	-0.003 (-0.011, 0.005)	0.49	-0.003 (-0.010, 0.004)	0.44
Perinatal mortality	-0.005 (-0.008, -0.002)	0.00	-0.004 (-0.007, -0.001)	0.002	-0.004 (-0.006, -0.001)	0.002
Preterm birth or low birth weight	0.011 (-0.007, 0.028)	0.23	0.005 (-0.010, 0.019)	0.53	0.004 (-0.008, 0.017)	0.49
Preterm birth (< 37 weeks)	0.011 (-0.005, 0.028)	0.18	0.006 (-0.008, 0.020)	0.40	0.006 (-0.006, 0.018)	0.37
Low birth weight (< 2500 grams)	0.003 (-0.009, 0.015)	0.63	-0.001 (-0.011, 0.008)	0.77	-0.002 (-0.011, 0.006)	0.61

Abbreviations: CI = confidence interval; OBGYN = obstetrician-gynecologist; MFM = maternal-fetal medicine.

Notes:

1. Sample includes all individuals with an expected date of delivery within 120 days of her 35th birthday. Individuals with an expected date of delivery within 7 days of the 35th birthday were excluded. Individuals with a low-risk pregnancy include all individuals without a diagnosis code for pregestational diabetes, chronic hypertension, obesity, or multiple gestation.

2. All regressions control for zip-code characteristics (percent white, percent Hispanic, median household income, and whether the zip code is urban) and county-level characteristics (any hospital with neonatal intensive care unit and OBGYNs per 10,000 deliveries). All regressions include state of residence, year, and month of delivery fixed effects.

© 2021 Geiger CK et al. *JAMA Health Forum.*

eTable 10. Unadjusted Regression Results

	Full Sample (N = 51,290)		Subgroup with Low-Risk Pregnancy (N = 40,472)	
	Unadjusted		Unadjusted	
**Outcomes**	**Coefficient (95% CI)**	**P-value**	**Coefficient (95% CI)**	**P-value**
Prenatal Care Services				
Total OBGYN visits	0.210 (-0.048, 0.468)	0.11	0.268 (0.012, 0.525)	0.04
Any MFM visit	0.043 (0.022, 0.063)	<0.001	0.052 (0.029, 0.075)	<0.001
Total ultrasounds	0.204 (0.040, 0.369)	0.02	0.235 (0.081, 0.389)	0.003
Any detailed ultrasound	0.156 (0.136, 0.176)	<0.001	0.160 (0.137, 0.182)	<0.001
Any antepartum fetal surveillance	0.048 (0.027, 0.069)	<0.001	0.059 (0.035, 0.082)	<0.001
Non-stress test	0.033 (0.012, 0.054)	0.002	0.039 (0.016, 0.062)	0.001
Biophysical profile	0.036 (0.018, 0.055)	<0.001	0.044 (0.024, 0.064)	<0.001
Any aneuploidy screening	0.010 (-0.008, 0.027)	0.29	0.006 (-0.014, 0.026)	0.58
Serum analyte	-0.040 (-0.060, -0.021)	<0.001	-0.046 (-0.068, -0.024)	<0.001
Cell-free DNA test	0.094 (0.080, 0.108)	<0.001	0.087 (0.071, 0.103)	<0.001
Invasive genetic test	0.011 (0.003, 0.018)	0.004	0.007 (-0.001, 0.015)	0.10
Maternal and Newborn Outcomes				
Any severe maternal morbidity	-0.003 (-0.011, 0.005)	0.45	-0.003 (-0.011, 0.005)	0.50
Perinatal mortality	-0.004 (-0.008, 0.000)	0.04	-0.004 (-0.007, -0.001)	0.002
Preterm birth or low birth weight	0.004 (-0.010, 0.018)	0.58	0.005 (-0.010, 0.019)	0.51
Preterm birth (< 37 weeks)	0.006 (-0.008, 0.020)	0.47	0.006 (-0.008, 0.020)	0.39
Low birth weight (< 2500 grams)	0.003 (-0.007, 0.013)	0.57	-0.001 (-0.011, 0.008)	0.80
Abbreviations: CI = confidence interval; OBGYN = obstetrician-gynecologist; MFM = maternal-fetal medicine.				
**Notes:**				
1. Sample includes all individuals with an expected date of delivery within 120 days of her 35th birthday. Individuals with an expected date of delivery within 7 days of the 35th birthday were excluded.				
2. Individuals with a low-risk pregnancy include all individuals without a diagnosis code for pregestational diabetes, chronic hypertension, obesity, or multiple gestation.				
3. All regressions include state of residence, year, and month of delivery fixed effects.				

© 2021 Geiger CK et al. *JAMA Health Forum.*

eTable 11. Placebo Regression Results Below Age 35

	**Full sample - Adjusted**							
	**34 Years and 4 Months (N = 43,806)**		**34 Years and 5 Months (N = 43,375)**		**34 Years and 6 Months (N = 42,896)**		**34 Years and 7 Months (N = 42,555)**	
**Outcomes**	**Coeff (CI)**	**P-value**	**Coeff (CI)**	**P-value**	**Coeff (CI)**	**P-value**	**Coeff (CI)**	**P-value**
Prenatal care services								
Total OB/GYN visits	0.023 (-0.215, 0.261)	0.848	-0.123 (-0.357, 0.112)	0.306	0.080 (-0.154, 0.313)	0.503	0.263 (0.024, 0.503)	0.031*
Any MFM visit	-0.020 (-0.039, -0.001)	0.041*	0.010 (-0.009, 0.029)	0.309	0.005 (-0.015, 0.024)	0.646	0.001 (-0.019, 0.020)	0.928
Total ultrasounds	-0.023 (-0.169, 0.123)	0.759	-0.051 (-0.194, 0.093)	0.487	0.054 (-0.091, 0.199)	0.467	0.113 (-0.034, 0.259)	0.131
Any detailed ultrasound	-0.009 (-0.029, 0.010)	0.342	0.003 (-0.017, 0.022)	0.788	0.006 (-0.014, 0.026)	0.542	0.001 (-0.019, 0.020)	0.940
Any antepartum surveillance	0.000 (-0.020, 0.019)	0.975	0.011 (-0.009, 0.031)	0.274	-0.003 (-0.023, 0.017)	0.789	0.001 (-0.019, 0.021)	0.912
Non-stress test	-0.001 (-0.021, 0.018)	0.887	0.017 (-0.003, 0.036)	0.092	-0.001 (-0.020, 0.019)	0.952	0.016 (-0.003, 0.036)	0.106
Biophysical profile	-0.003 (-0.020, 0.014)	0.719	0.014 (-0.004, 0.031)	0.121	-0.006 (-0.024, 0.011)	0.487	0.000 (-0.018, 0.017)	0.991
Any aneuploidy screening	0.009 (-0.009, 0.026)	0.343	0.001 (-0.017, 0.018)	0.951	-0.005 (-0.023, 0.012)	0.556	0.010 (-0.008, 0.028)	0.259
Serum analyte	0.007 (-0.011, 0.026)	0.430	-0.004 (-0.022, 0.015)	0.709	-0.008 (-0.026, 0.011)	0.415	0.008 (-0.011, 0.027)	0.393
Cell-free DNA test	0.001 (-0.011, 0.013)	0.834	0.003 (-0.009, 0.016)	0.583	0.002 (-0.010, 0.015)	0.725	0.009 (-0.004, 0.022)	0.165
Invasive genetic test	0.001 (-0.005, 0.007)	0.756	-0.001 (-0.008, 0.005)	0.743	0.000 (-0.007, 0.006)	0.911	-0.001 (-0.008, 0.005)	0.672
Maternal and newborn outcomes								
Any severe maternal morbidity	0.005 (-0.003, 0.012)	0.225	0.001 (-0.006, 0.008)	0.746	0.001 (-0.006, 0.008)	0.815	0.004 (-0.003, 0.012)	0.260
Perinatal mortality (GA ≥ 28 weeks, within 7 days)	-0.001 (-0.005, 0.002)	0.446	0.001 (-0.003, 0.004)	0.719	0.002 (-0.002, 0.006)	0.389	-0.002 (-0.006, 0.002)	0.311
Preterm delivery or low birth weight	-0.014 (-0.027, -0.001)	0.036*	0.006 (-0.007, 0.019)	0.349	0.006 (-0.008, 0.020)	0.384	0.004 (-0.010, 0.018)	0.585
Preterm (< 37 weeks)	-0.014 (-0.027, -0.001)	0.037*	0.008 (-0.005, 0.021)	0.222	0.005 (-0.009, 0.018)	0.478	0.003 (-0.010, 0.016)	0.663

© 2021 Geiger CK et al. *JAMA Health Forum.*

Low birth weight (< 2500 grams)	-0.005 (-0.014, 0.004)	0.285	-0.001 (-0.010, 0.008)	0.866	0.007 (-0.003, 0.016)	0.187	0.000 (-0.010, 0.009)	0.940
**Notes:**								
1. For the placebo tests, the cutoff for the regression discontinuity was changed from age 35 to the date specified in each column. Sample includes all mothers with an expected delivery date within 120 days of the specified birthdate for each placebo test.								
2. All adjusted regressions for the full sample and the subgroup in the lowest income zip codes controlled for individual-level characteristics (chronic diabetes, chronic hypertension, obesity, and multiple gestation) and zip code characteristics (percent white, percent Hispanic, median household income, and whether the zip code is urban), and county-level characteristics (any hospital with neonatal intensive care unit and OB/GYNs per 10,000 deliveries). All adjusted regressions also included state, year, and month of delivery fixed effects.								

© 2021 Geiger CK et al. *JAMA Health Forum.*

eTable 12. Placebo Regression Results Above Age 35

	**Full sample - Adjusted**							
	**35 Years and 5 Months (N = 38,335)**		**35 Years and 6 Months (N = 37,664)**		**35 Years and 7 Months (N = 37,177)**		**35 Years and 8 Months (N = 36,868)**	
**Outcomes**	**Coeff (CI)**	**P-value**	**Coeff (CI)**	**P-value**	**Coeff (CI)**	**P-value**	**Coeff (CI)**	**P-value**
Prenatal care services								
Total OB/GYN visits	-0.005 (-0.261, 0.251)	0.972	0.098 (-0.160, 0.355)	0.459	-0.035 (-0.302, 0.231)	0.795	0.013 (-0.254, 0.280)	0.925
Any MFM visit	0.017 (-0.004, 0.037)	0.112	-0.004 (-0.025, 0.017)	0.713	-0.008 (-0.029, 0.013)	0.469	-0.005 (-0.026, 0.016)	0.666
Total ultrasounds	0.114 (-0.046, 0.275)	0.161	-0.011 (-0.169, 0.146)	0.887	0.015 (-0.146, 0.176)	0.856	0.028 (-0.137, 0.193)	0.738
Any detailed ultrasound	0.041 (0.021, 0.060)	0.000***	0.006 (-0.013, 0.026)	0.530	-0.023 (-0.042, -0.003)	0.024*	0.005 (-0.015, 0.025)	0.630
Any antepartum surveillance	0.029 (0.009, 0.050)	0.005**	-0.003 (-0.024, 0.017)	0.748	0.001 (-0.020, 0.022)	0.907	0.008 (-0.013, 0.029)	0.455
Non-stress test	0.032 (0.011, 0.053)	0.003**	-0.009 (-0.030, 0.012)	0.414	0.000 (-0.022, 0.021)	0.989	0.012 (-0.010, 0.033)	0.282
Biophysical profile	0.019 (0.000, 0.038)	0.055	0.002 (-0.017, 0.021)	0.836	0.000 (-0.019, 0.020)	0.984	-0.004 (-0.024, 0.015)	0.656
Any aneuploidy screening	0.004 (-0.013, 0.022)	0.628	0.005 (-0.013, 0.023)	0.578	-0.015 (-0.033, 0.003)	0.100	-0.003 (-0.021, 0.015)	0.771
Serum analyte	-0.001 (-0.021, 0.019)	0.907	0.005 (-0.015, 0.025)	0.638	-0.006 (-0.027, 0.014)	0.535	-0.011 (-0.032, 0.009)	0.283
Cell-free DNA test	-0.004 (-0.019, 0.011)	0.608	-0.004 (-0.019, 0.010)	0.548	0.000 (-0.015, 0.014)	0.952	-0.004 (-0.019, 0.011)	0.606
Invasive genetic test	0.003 (-0.006, 0.012)	0.508	0.001 (-0.008, 0.010)	0.832	0.000 (-0.009, 0.009)	0.932	0.004 (-0.005, 0.014)	0.362
Maternal and newborn outcomes								
Any severe maternal morbidity	-0.003 (-0.011, 0.006)	0.525	-0.002 (-0.010, 0.006)	0.695	0.003 (-0.005, 0.011)	0.431	0.002 (-0.005, 0.010)	0.546
Perinatal mortality (GA ≥ 28 weeks, within 7 days)	0.000 (-0.005, 0.004)	0.914	-0.001 (-0.006, 0.003)	0.489	0.001 (-0.004, 0.005)	0.760	0.000 (-0.004, 0.004)	0.952
Preterm delivery or low birth weight	0.014 (-0.001, 0.029)	0.059	-0.006 (-0.021, 0.009)	0.415	-0.005 (-0.020, 0.009)	0.480	-0.011 (-0.026, 0.004)	0.134

© 2021 Geiger CK et al. *JAMA Health Forum.*

Preterm (< 37 weeks)	0.014 (0.000, 0.028)	0.058	-0.005 (-0.019, 0.010)	0.536	-0.003 (-0.018, 0.011)	0.644	-0.011 (-0.026, 0.003)	0.124
Low birth weight (< 2500 grams)	0.004 (-0.007, 0.015)	0.457	-0.003 (-0.014, 0.007)	0.566	-0.005 (-0.016, 0.005)	0.331	0.001 (-0.009, 0.012)	0.836

Notes:

1. For the placebo tests, the cutoff for the regression discontinuity was changed from age 35 to the date specified in each column. Sample includes all mothers with an expected delivery date within 120 days of the specified birthdate for each placebo test.

2. All adjusted regressions for the full sample and the subgroup in the lowest income zip codes controlled for individual-level characteristics (chronic diabetes, chronic hypertension, obesity, and multiple gestation) and zip code characteristics (percent white, percent Hispanic, median household income, and whether the zip code is urban), and county-level characteristics (any hospital with neonatal intensive care unit and OB/GYNs per 10,000 deliveries). All adjusted regressions also included state, year, and month of delivery fixed effects.

© 2021 Geiger CK et al. *JAMA Health Forum.*

Diagnosis of Elderly Primigravida and/or Multigravida

In order to evaluate whether providers were recognizing individuals as being designated as AMA at the age 35 cutoff that we calculated, rather than during pregnancy, we examined diagnosis codes for “elderly primigravida and/or multigravida” during pregnancy. This diagnosis code should be used for individuals of AMA, defined as age 35 years or older on the expected date of delivery, and be consistent with our identification of individuals of AMA at the age 35 cutoff. Using the previously described RD methods, we ran the local linear regressions using an indicator for having a diagnosis code for elderly primigravida and/or multigravida as the outcome (eTable 2). As shown in eFigure 5 and eTable 13, the use of this diagnosis code jumps substantially at the cutoff. Taken together with our results that also showed sharp increases in prenatal care services at the cutoff, this indicates that providers do frequently recognize AMA based on the age at the expected date of delivery consistent with our study design.

© 2021 Geiger CK et al. *JAMA Health Forum.*

eTable 13. Regression Results for Diagnosis Code for Elderly Primigravida and/or Multigravida During Pregnancy

	**Full Sample (N = 51,290)**		**Subgroup with Low-Risk Pregnancy (N = 40,472)**	
	**Adjusted**		**Adjusted**	
**Outcomes**	**Coefficient (95% CI)**	**P-value**	**Coefficient (95% CI)**	**P-value**
Elderly primigravida and/or multigravida	0.504 (0.487, 0.522)	<0.001	0.496 (0.476, 0.516)	<0.001
Abbreviations: CI = confidence interval				
**Notes:**				
1. Sample includes all individuals with an expected date of delivery within 120 days of her 35th birthday. Individuals with an expected date of delivery within 7 days of the 35th birthday were excluded.				
2. Individuals with a low-risk pregnancy include all individuals without a diagnosis code for pregestational diabetes, chronic hypertension, obesity, or multiple gestation.				
3. All regressions control for individual-level characteristics (pregestational diabetes, chronic hypertension, obesity, multiple gestation), zip-code characteristics (percent white, percent Hispanic, median household income, and whether the zip code is urban), and county-level characteristics (any hospital with neonatal intensive care unit and OBGYNs per 10,000 deliveries). All regressions include state of residence, year, and month of delivery fixed effects.				

© 2021 Geiger CK et al. *JAMA Health Forum.*

eFigure 5. Diagnosis Codes for Elderly Primigravida and/or Multigravida During Pregnancy by Weeks Relative to Age 35 on Expected Date of Delivery

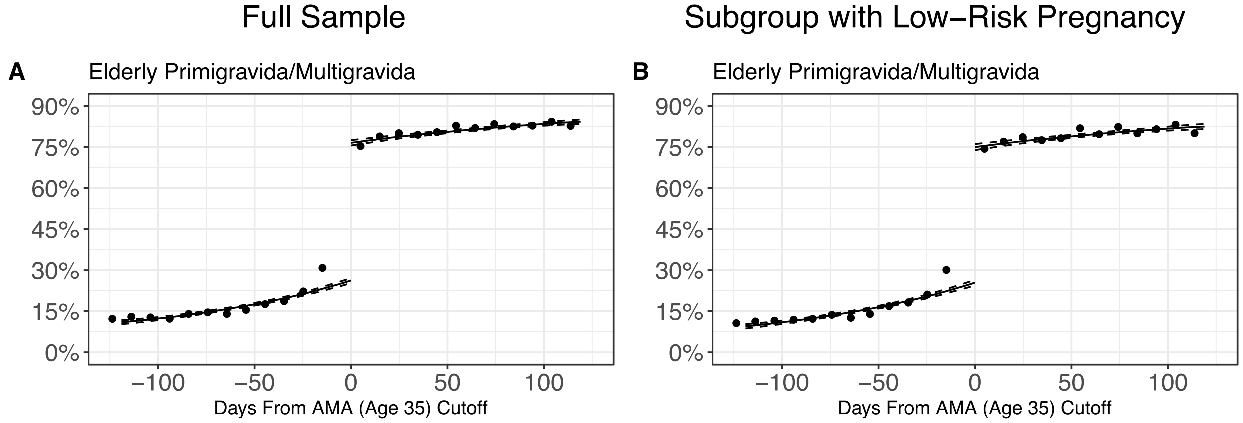

Legend: The figure shows adjusted local linear regression results for the regression discontinuity analyses (solid lines) and the 95% confidence intervals (dashed lines). The points in the figure show the binned unadjusted outcomes plotted by the running variable (i.e., the number of days between the expected date of delivery and 35th birthday). Panel A shows the results for the full sample (N=51,290) and panel B shows the results for the subgroup with a low-risk pregnancy (N=40,472). All individuals in the sample had an expected date of delivery within 120 days of their 35th birthday. As seen in the figure and the regression results, the AMA designation was associated with a significant increase in the proportion of individuals with a diagnosis code for "elderly primigravida/multigravida".

© 2021 Geiger CK et al. *JAMA Health Forum.*

eFigure 6. Changes in Rate of Induction of Labor During the Study Period

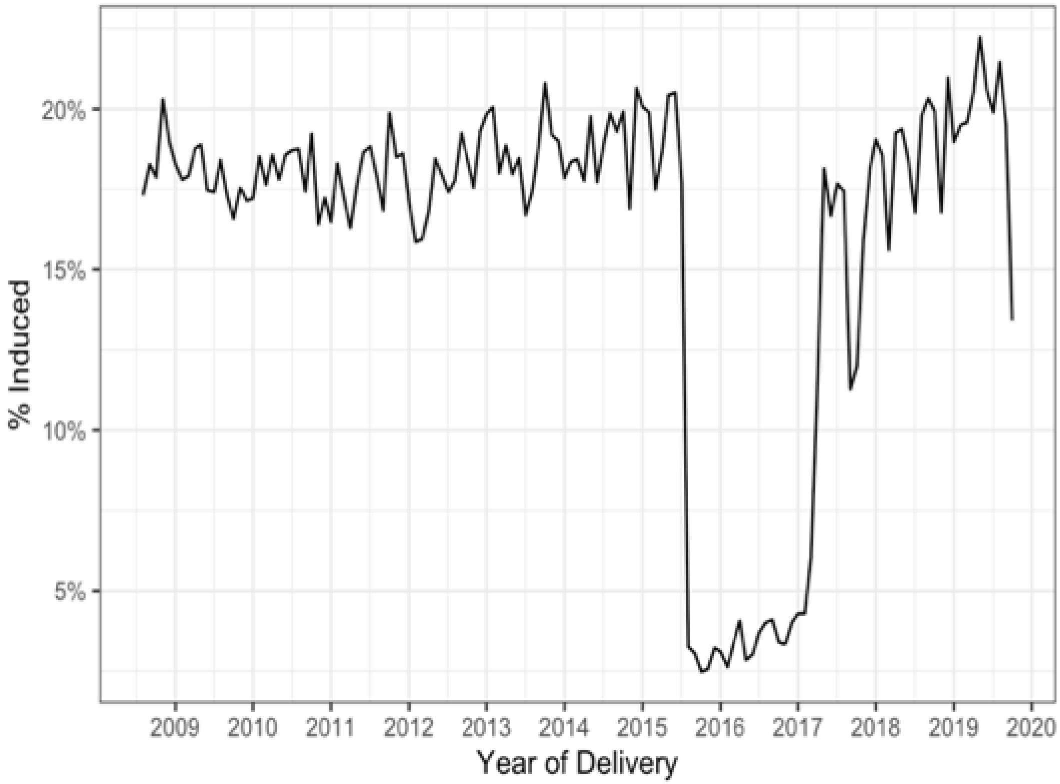

Legend: Shown is the rate of induction of labor among all individuals in the full sample by the year of delivery. The sample includes all individuals with an expected date of delivery within 120 days of her 35th birthday. Induction of labor was identified using ICD-9 diagnosis (659.0, 659.1) and procedure codes (73.4, 73.01, 73.1) and ICD-10 diagnosis (O61) and procedure codes (3E033VJ, 3E0P7GC, 3E0P7VZ, 0U7C7ZZ, 0U7C7DZ, 3E0P3VZ).

In this study, we were unable to explore changes in induction of labor at the age 35 cutoff due to the limitations of coding for the procedure in claims data. Induction of labor is included in the global billing codes for delivery so there is a lack of any financial incentive to code for induction in claims data, which would reduce our ability to accurately identify inductions. In addition, during our study period, there was a sharp decline in coding for induction of labor at the time of the switch from ICD-9 to ICD-10 codes on October 1, 2015. As shown in eFigure 6, inductions dropped sharply in October 2015 and did not rebound for over one year.

© 2021 Geiger CK et al. *JAMA Health Forum.*
